# Ferrostatin-1 protects against early sepsis-induced acute lung injury by suppressing lipid peroxidation–driven NINJ1-mediated DAMP release and neutrophil activation

**DOI:** 10.1016/j.redox.2026.104004

**Published:** 2026-01-06

**Authors:** Fang Xiao, Donghua Li, Miao Yu, Yunfeng Zhu, Guorong Huang, Zhilei Huang, Yufang Wang, Jialin Li, Dongmei Zhong, Huan Ma, Kunyu Liao, Yongshan Liu, Yalin Zhang, Xiangdong Guan, Changjie Cai, Jing Tang, Tianqin Peng, Fu-Li Xiang, Jie Xu

**Affiliations:** a Department of Critical Care Medicine, The First Affiliated Hospital of Sun Yat-sen University, Guangzhou, China; b Institute of Precision Medicine, The First Affiliated Hospital of Sun Yat-sen University, Guangzhou, China; c NHC Key Laboratory of Assisted Circulation and Vascular Diseases, Sun Yat-sen University, Guangzhou, China; d National‐Guangdong Joint Engineering Laboratory for Diagnosis and Treatment of Vascular Diseases, Guangzhou, China; e The Department of Anesthesiology, Affiliated Hospital of Guangdong Medical University, Zhanjiang, Guangdong, China; f Departments of Medicine and Pathology and Laboratory Medicine, Western University, London Health Sciences Centre Research Institute, Canada

**Keywords:** Cardiopulmonary disease, Ferrostatin-1, Lipid-peroxidation associated cell death, Plasma membrane damage, DAMP release, Neutrophil, Inflammatory cytokine, Acute lung injury, Sepsis

## Abstract

Sepsis-induced acute lung injury (ALI) is a critical condition driven by neutrophil-dominated inflammation, lytic cell death and the subsequent DAMP release, etc. We tested whether the radical-trapping antioxidant Ferrostatin-1 (Fer-1) interrupts lipid peroxidation induced DAMP release and limits early lung injury in sepsis. We found that Fer-1 improved survival, preserved alveolar architecture, reduced lung-injury scores, and suppressed pulmonary inflammatory cytokine expression in a murine cecal ligation and puncture (CLP) model. Lung tissue RNA-sequencing showed that Fer-1 attenuated the CLP-induced inflammatory and chemotaxis transcriptome and significantly reduced neutrophil infiltration. *In vitro*, Fer-1 protected cells from lipid peroxidation–induced lytic death and impaired the release of large DAMPs associated with NINJ1 pathway, indicated Fer-1 acts upstream of NINJ1 to preserve membrane integrity. Fer-1 also directly lowered lipid peroxidation and reduced lipopolysaccharide (LPS)–induced IL-1β and IL-6 transcription and secretion in neutrophils, an effect reversed by pharmacological JNK/p38 activation. Together, our results indicate that Fer-1 functions as a dual-action modulator that prevents DAMP release and blunts neutrophil-driven inflammation escalation, thereby interrupting the lipid peroxidation–NINJ1–DAMP release axis, and mitigating early septic ALI.

## Highlights

• Ferrostatin-1 (Fer-1) blunts early neutrophil infiltration and attenuates lung injury in the sepsis-induced acute lung injury.
• Fer-1 reduces NINJ1-mediated DAMP release by maintaining plasma membrane integrity.
• Fer-1 directly suppresses IL-1β/IL-6 production via inhibition of lipid peroxidation-induced JNK/p38 pathways.

## 1 Introduction

Sepsis, a life-threatening organ dysfunction caused by a dysregulated host response to infection, remains a leading cause of mortality in intensive care units worldwide [1]. The lung is one of the most vulnerable organs during sepsis, frequently leading to acute lung injury (ALI) or its more severe form, acute respiratory distress syndrome (ARDS) [2]. The pathogenesis of sepsis-induced ALI is complex, involving a cascade of events including uncontrolled inflammation, excessive oxidative stress, and programmed cell death, which collectively compromise the alveolar-capillary barrier and impair gas exchange [3,4]. Despite advances in critical care, effective pharmacological treatments targeting these underlying mechanisms are urgently needed.

The pathophysiology of septic acute lung injury (ALI) is characterized by a multi-step inflammatory cascade driven by a feed-forward amplification loop [4,5]. Initially, sepsis-induced systemic hypoperfusion and ischemia/ROS induce programmed necrosis (necroptosis) in pulmonary epithelial and endothelial cells [[6], [7], [8]]. This process results in the release of damage-associated molecular patterns (DAMPs), such as dsDNA and HMGB1 [6]. These DAMPs subsequently activate receptors on resident alveolar macrophages, initiating the expression of chemokines, including CXCL1 and CXCL2 [9,10]. Circulating neutrophils are then recruited to the lung along this established chemokine gradient [11]. Following extravasation, these neutrophils become fully activated and release cytotoxic substances through degranulation, ROS production, and NETosis to clear pathogens [11,12]. This defensive response can concurrently cause collateral lung tissue damage and promote microvascular thrombosis. Simultaneously, the activated neutrophils release a secondary, significant quantity of pro-inflammatory cytokines (e.g., IL-1β, IL-6, and TNF-α) and additional chemokines [13,14]. These mediators act in an autocrine and paracrine fashion to recruit more neutrophils, which in turn perpetuate ROS generation and proteolytic damage. This self-sustaining inflammatory cycle can escalate the initial, localized containment response into systemic cytokine overproduction and high-level neutrophil infiltration [15]. This phenomenon directly mediates the alveolar-capillary barrier disruption and refractory pulmonary edema characteristic of ARDS [16]. Therefore, therapeutic strategies targeting either the initial DAMP release or the subsequent inflammatory amplification cycle may hold therapeutic potential.

Ferrostatin-1 (Fer-1) is a potent small-molecule inhibitor of lipid peroxidation that functions as a lipophilic radical-trapping antioxidant to prevent lipid-peroxidation–induced cell death [17]. The protective actions of Fer-1 have been demonstrated across multiple organ-injury models, including LPS-induced acute lung injury, ischemia–reperfusion injury, and ventilator-induced lung injury, primarily through suppression of oxidative stress and restoration of GPX4/SLC7A11-dependent antioxidant defenses [[18], [19], [20], [21]]. Importantly, recent studies using the cecal ligation and puncture (CLP) model of polymicrobial sepsis show that Fer-1 confers organ-specific protection by mitigating lipid-peroxidation-associated tissue dysfunction [[22], [23], [24]]. In sepsis-associated encephalopathy, Fer-1 decreased neuronal lipid peroxidation, preserved BBB integrity, and improved neurological performance via Nrf2/HO-1 activation [22]. Fer-1 also attenuated intestinal barrier injury, reducing bacterial translocation and restoring tight-junction integrity after CLP [23], and alleviated renal lipid-peroxidation induced cell death in sepsis-associated acute kidney injury [24]. These findings collectively indicate that lipid-peroxidation contributes to multi-organ dysfunction in sepsis. Notably, most studies evaluate Fer-1's effects around 24 h after CLP, when organ damage, cytokine storm, and lipid-peroxidation-associated tissue dysfunction signatures are already well established. Consequently, the very early pathological processes that initiate sepsis-associated tissue injury, including large DAMP release, lipid membrane disruption, neutrophil priming, and early organ dysfunction, remain poorly understood. In our recent work, we demonstrated that in CLP-induced cardiomyopathy, Fer-1 reduced neutrophil infiltration, inflammatory chemokines, and improved cardiac functional strain parameters [25] 6h after CLP.

Therefore, this study was designed to investigate the hypothesis that Fer-1 protects against sepsis-induced ALI by inhibiting the initial lipid-peroxidation-related cell membrane injury and DAMP-driven neutrophil activity that precede overt sepsis-associated ALI. We utilized CLP mouse model to elucidate the effects of Fer-1 on early lung injury, immune cell infiltration, and inflammation in sepsis.

## 2 Materials and methods

### 2.1 Animals and CLP model

All animal experiments were carried out in accordance with procedures approved by the Institutional Animal Care and Use Committee of Sun Yat-sen University (SYSU-IACUC-2024-002534). Male C57BL/6J mice of 8∼12 weeks old were used in this research and purchased from Guangzhou Ruige Biological Technology Co., LTD. Before experiment, animals were housed under specific pathogen-free conditions in a 12 h light/dark cycle and temperature-controlled room (19–25 °C) with free access to food and water.

As described previously [2,25] 2h before CLP-induced sepsis, mice of the CLP + Fer-1 group were i.p. injected with Fer-1 (Selleck Chemicals, S7243, 5 mg/kg), and CLP group were i.p. injected with vehicle for Fer-1. Mice were anesthetized with inhalation of 2 % isoflurane and 1 L/min O_2_ under sterile conditions. The peritoneal cavity was cut open, the cecum was ligated at 1 cm from the distal end, and the distal cecum was punctured for a single hole with 16G needle to induce sepsis. A small amount of fecal was squeezed out of the hole of the cecum before the cecum was returned to the peritoneal cavity. The incision was sutured with 5-0 silk suture. Subsequently, fluid resuscitation was performed by subcutaneous injection of 1 mL of 0.9 % sterile saline. Sham-operated mice underwent the same surgical procedures, including laparotomy, but without cecal ligation or puncture.

### 2.2 Experimental design and SAGER guidelines

In accordance with SAGER (Sex and Gender Equity in Research) guidelines, we report that this study was conducted exclusively in male mice aged 8–12 weeks. This single-sex design was chosen to minimize biological variability associated with the estrous cycle in females and to avoid the known confounding effects of female sex hormones in sepsis injury models, thereby reducing the total number of animals required to achieve statistical power. We acknowledge that this approach limits the direct generalizability of our findings. The role of Fer-1 in sepsis-induced ALI may differ in females, and future studies are warranted to investigate potential sex-specific mechanisms. For *in vivo* experiments, mice were randomly allocated to treatment groups. Key outcome assessments, including the histology analysis and the quantification of DAMP release, were performed by investigators who were blinded to the treatment and surgical group of the animals.

### 2.3 Neutrophil isolation and stimulation

Mice were i.p. injected with 1 mL 3 % thioglycollate broth medium (Solarbio, LA8740). After 4–6 h, mice were euthanized, and neutrophils were collected by peritoneal lavage with 10 mL ice-cold RPMI-1640 medium containing 1 % FBS [26]. Neutrophils were seeded and treated with 50 μM Ferrostatin-1 (Selleck, S7243) for 1 h followed by 50 ng/mL Lipopolysaccharide originated from *E. coli* 055: B5 (Meilunbio, MB5198) for 4 h. Cells were collected for RNA isolation or immunoblot and supernatant was collected for measurement of released cytokines. For secretion of IL-1β, 5 mM ATP was added 1 h before termination. 100 ng/mL Anisomycin (Selleck, S7409) was added 30 min before adding LPS.

### 2.4 Statistics

All quantitative data are presented as mean ± SEM. Statistical analyses were performed using GraphPad Prism software (v9.0, GraphPad Software). For comparisons between two groups with normally distributed data, an unpaired, two-tailed Student's t-test was used. For comparisons between more than two groups with normally distributed data, a one-way or two-way Analysis of Variance (ANOVA) was performed, followed by Tukey's multiple comparisons post-hoc test. For data that were not normally distributed, the non-parametric Mann-Whitney *U* test (for two groups) or the Kruskal-Wallis test with Dunn's multiple comparisons test (for multiple groups) was used. Survival curves were generated using the Kaplan-Meier method and compared using the Log-rank (Mantel-Cox) test. A P-value <0.05 was considered statistically significant. The specific tests used for each experiment are detailed in the figure legends. Please see detailed methodology in **Supplemental Methods**.

## 3 Results

### 3.1 Fer-1 improves CLP-induced sepsis survival and attenuates sepsis-induced lung injury

**Fig. 1:**
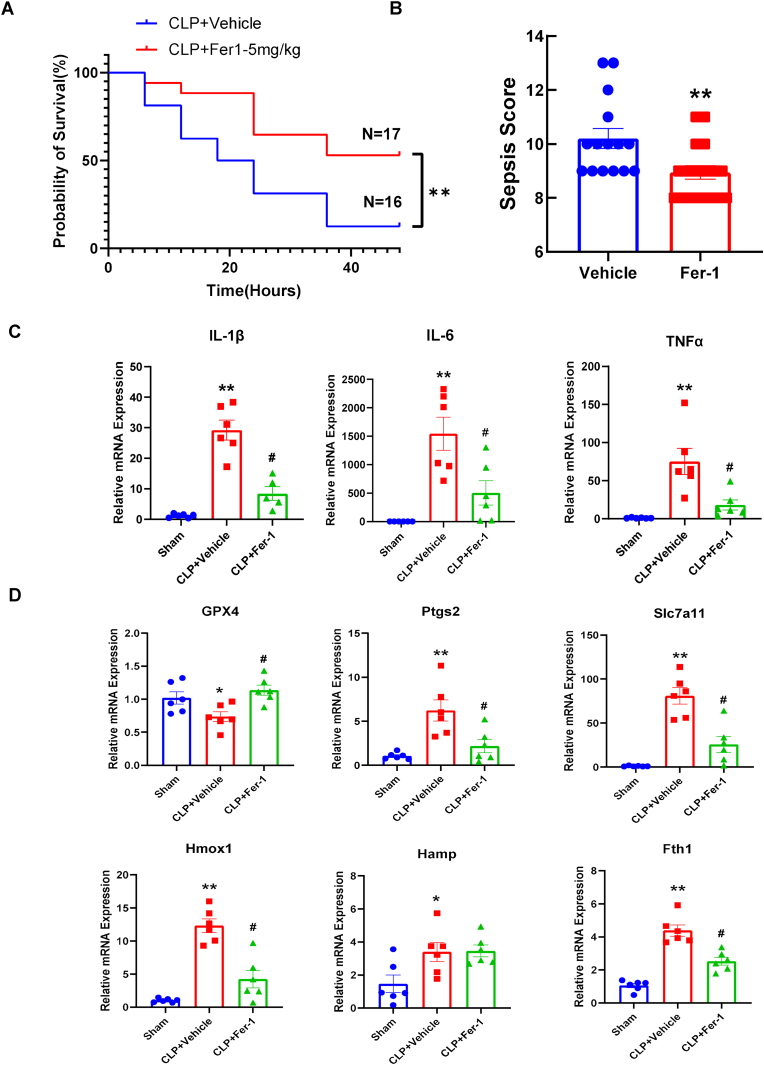
**Fer-1 improves survival and attenuates pulmonary inflammation and ferroptosis-associated gene expression in septic mice.** (A) Kaplan-Meier survival curve for 48 h in CLP mice treated with Vehicle (N = 16) or Fer-1 (5 mg/kg, N = 17). ∗∗*P* < 0.01 vs. Vehicle (B) Sepsis severity score in Vehicle and Fer-1 treated mice. ∗∗*P* < 0.01 vs. Vehicle analyzed by unpaired, two-tailed Student's t-test (C) Relative mRNA expression of proinflammatory cytokines *IL-1β*, *IL-6*, and *TNFα* in lung tissue 6 h post-CLP from Sham, CLP + Vehicle, and CLP + Fer-1 groups. (D) Relative mRNA expression of ferroptosis and iron-metabolism-related genes (*GPX4, Ptgs2, Slc7a11, Hmox1, Hamp,* and *Fth1*) in lung tissue 6h post-CLP. Data are presented as mean ± SEM. N = 5–6, ∗*P* < 0.05, ∗∗*P* < 0.01 vs. Sham; #*P* < 0.05 vs. CLP + Vehicle analyzed by one-way ANOVA with Tukey's post-hoc test in (C) and (D).

First, we established and characterized the cecal ligation and puncture (CLP) model in house. Sepsis induced by CLP resulted in significant 48-h mortality, with survival dropping to approximately 40 %, compared to 100 % survival in sham-operated mice (Supplemental Fig. 1A). At 24 h post-surgery, CLP mice exhibited severe acute lung injury (ALI), which was confirmed histologically by massive inflammatory cell infiltration, alveolar septal thickening, and edema compared to the normal lung architecture of the sham group (Supplemental Fig. 1B). Consistent with our previous findings [25], early cardiac dysfunction at 6 h post-CLP showed a significant reduction in the E/A ratio, as well as decreased global longitudinal and radial strain (Supplemental Fig. 1C and D). Pre-treatment with Fer-1 (5 mg/kg) significantly improved the 48-h survival rate of CLP mice and significantly lowered the clinical sepsis score (Fig. 1A and B).

To investigate the early pathological changes in sepsis-induced ALI before mortality starts, we analyzed lung tissue 6 h after CLP. The CLP model induced significant systemic iron dysregulation, as shown by significantly elevated levels of both serum Fe^2+^ and total iron (Supplemental Fig. 2A). A trend in increased iron staining within the CLP lung tissue was observed but did not reach the significant level (Supplemental Fig. 2B). Histological analysis of lung tissue at 6 h post-CLP demonstrated that Fer-1 treatment markedly attenuated ALI, preserving alveolar structure as shown by lung injury score quantification (Supplemental Fig. 2C). At the transcriptional level, CLP dramatically induced transcriptional upregulation of key proinflammatory cytokines in the lung, including IL-1β, IL-6, and TNFα (Fig. 1C), which were all significantly attenuated by Fer-1 pre-treatment. We next examined the genes associated with lipid oxidation and iron dysregulation, the reported target pathways of Fer-1 [27]. *Gpx4*, *Ptgs2*, *Slc7a11*, *Hmox1*, and *Fth1* gene modestly but significantly changed at 6h after CLP, while Fer-1 treatment effectively countered this dysregulation (Fig. 1D). These data demonstrated the early protective role of Fer-1 in CLP induced acute lung injury and showed that Fer-1 can regulate pro-inflammatory cytokine and ferroptosis-related gene expression in the lung tissue.

### 3.2 Fer-1 treated septic lung shows a transcriptome profile of attenuated proinflammatory cytokine and chemokine signature

To understand the molecular mechanisms behind Fer-1's protective effects, we performed RNA-sequencing on lung tissue 6 h post-CLP. As expected, CLP induced a massive transcriptomic shift compared to sham controls, with 2268 genes upregulated and 2212 genes downregulated (Supplemental Fig. 3A–C). Comparing CLP + Fer-1 to CLP + Vehicle, we identified 1185 upregulated and 575 downregulated genes, demonstrating that Fer-1 actively reprograms the septic response (Supplemental Fig. 3D–F).

**Fig. 2:**
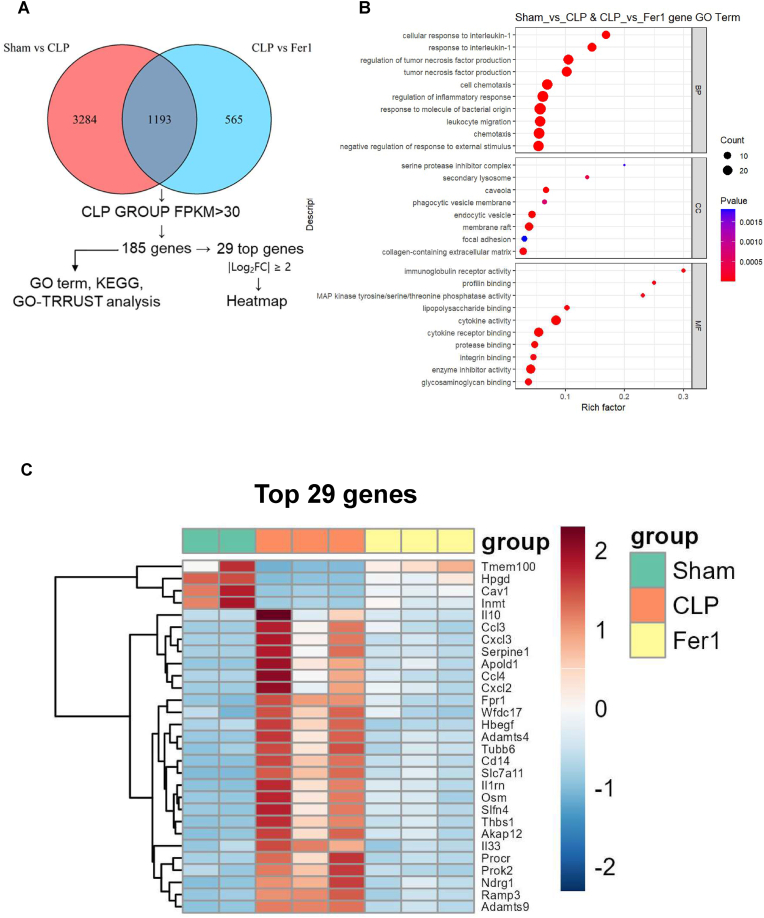
Fer-1 reverses the sepsis-induced transcriptomic signature for inflammation and chemotaxis in lung tissue 6h after CLP. (A) Venn diagram illustrating the overlap of differentially expressed genes (DEGs) identified in Sham vs CLP (3284 unique) and CLP vs CLP + Fer-1 (565 unique) comparisons. 185 enriched genes were selected from 1193 DEGs common to both datasets. (B) Gene Ontology (GO) enrichment analysis of the 185 overlapping DEGs, showing top terms for Biological Process (BP), Cellular Component (CC), and Molecular Function (MF). Enriched terms are related to cell chemotaxis, cytokine signaling, and chemokine activity. (C) Heatmap visualizing relative expression of representative DEGs from the 29 top overlap genes, comparing Sham, CLP, and Fer-1 groups. Red indicates high expression, blue indicates low expression.

To identify the core pathways reversed by Fer-1, we compared the differentially expressed genes (DEGs) between the “Sham vs CLP” and “CLP vs Fer-1″ groups. A Venn diagram analysis revealed a crucial overlap of 1193 DEGs that were induced by CLP and subsequently reversed by Fer-1 (Fig. 2A). These 1193 genes were filtered for high expression in CLP group (FPKM >30), resulting in a core set of 185 genes for further analysis. To understand the biological functions of these 185 core genes, we performed GO and KEGG pathway enrichment analyses. The GO term analysis (Fig. 2B) showed that the genes were highly enriched in Biological Processes (BP) related to inflammation, including “cellular response to interleukin-1″, “regulation of tumor necrosis factor production”, “regulation of inflammatory response”, “cell chemotaxis”, and “leukocyte migration”. Enriched Molecular Functions (MF) included “cytokine activity”, “cytokine receptor binding”, and “lipopolysaccharide binding”. KEGG pathway analysis (Supplemental Fig. 4A) confirmed this strong inflammatory signature. The most significantly enriched pathways included the “IL-17 signaling pathway”, “TNF signaling pathway”, “NF-kappa B signaling pathway”, “Toll-like receptor signaling pathway”, and “Chemokine signaling pathway”. Notably, the “Ferroptosis” pathway was also significantly enriched, supporting the mechanism of action for Fer-1. Finally, a GO-TRRUST analysis identified the key transcription factors (TFs) potentially regulating this gene set (Supplemental Fig. 4B). The analysis highlighted TFs central to inflammation, including *Nfkb1*, *Rela*, *Jun*, *Stat3*, *Egr1*, and *Hif1a*, suggesting that Fer-1 treatment may interfere with the activation of these pro-inflammatory transcriptional programs.

A heatmap of the top 29 DEGs (Fig. 2C) from the 185-gene list illustrates the expression pattern across the three groups. Pro-inflammatory chemokines and cytokines such as *Ccl3*, *Cxcl3*, *Ccl4*, *Cxcl2*, as well as *Cd14* and *Il1rn*, were highly upregulated in the CLP group (orange). This upregulation was markedly reversed in the Fer-1 treatment group (yellow), with expression levels returning closer to those of the Sham controls (green). As the initiation of chemotaxis and inflammation is largely associated with DAMP release, we generated a heatmap of the differentially expressed genes associated with DAMP release (Supplemental Fig. 4C) [28,29]. Key inflammatory mediators like *Tnf*, *Il6*, *S100a8*, *S100a9*, and the necroptosis/pyroptosis-related genes *Ripk3* and *Casp1* were all highly expressed in the CLP group but suppressed in both the Sham and Fer-1 groups. Remarkably, expression of *Ninj1*, the regulator for membrane rupture associated DAMP release recently identified by our group and others [[30], [31], [32], [33]], was upregulated in CLP group (Supplemental Fig. 4C).

**Fig. 3:**
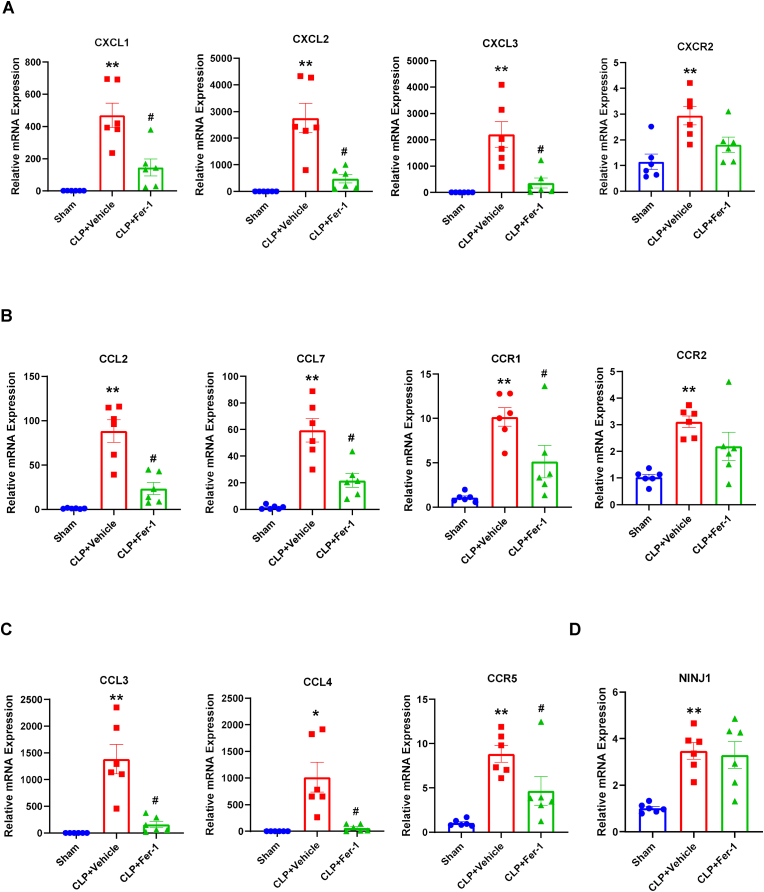
Fer-1 validation confirms broad suppression of chemokine and chemokine receptor expression in the lung tissue 6h after CLP. Relative mRNA expression of chemokines and their receptors in lung tissue 6 h post-CLP in Sham, CLP + Vehicle, and CLP + Fer-1 groups. (A) CXC-family chemokines CXCL1, CXCL2, CXCL3, and receptor CXCR2. (B) CC-family chemokines CCL2, CCL7, and receptors CCR2 and CCR1. (C) CC-family chemokines CCL3, CCL4, and receptor CCR5. (D) Relative mRNA expression of NINJ1. Data are presented as mean ± SEM. N = 6, ∗*P* < 0.05, ∗∗*P* < 0.01 vs. Sham; #*P* < 0.05 vs. CLP + Vehicle analyzed by one-way ANOVA with Tukey's post-hoc test.

Based on the RNA-seq findings, we validated the expression of a wide array of chemokines and their receptors using qPCR. This analysis confirmed a broad and potent suppression of the chemokine gene expression in the lung by Fer-1. The expression of the potent neutrophil chemoattractant *Cxcl1*, *Cxcl2*, and *Cxcl3* were all massively upregulated by CLP, and this induction was significantly suppressed by Fer-1 treatment (Fig. 3A). A similar and significant suppression was observed for the CC-family chemokines, including *Ccl2*, *Ccl3*, *Ccl4*, *Ccl7*, *Ccl11*, *Ccl17*, and *Ccl22* (Fig. 3B and C, Supplemental Fig. 5). Fer-1 also downregulated the expression of the corresponding chemokine receptors. The CLP-induced upregulation of *Ccr1* and *Ccr5* was all significantly attenuated by Fer-1 treatment (Fig. 3B and C). Interestingly, while genes like *Ccr2*, *Cxcr2*, *Ccl5* and *Cxcl5* were upregulated by CLP, their expression was not significantly altered by Fer-1, suggesting a specific, rather than global, anti-inflammatory effect (Fig. 3A and B; Supplemental Fig. 5). Finally, we examined the expression of *Ninj1* (Ninjurin-1), a key mediator of lytic cell death-induced DAMP release. While *Ninj1* mRNA was significantly upregulated by CLP, this upregulation was not significantly reversed by Fer-1 treatment at the transcriptional level (Fig. 3D).

In summary, bulk tissue transcriptome analysis of septic lung tissue at 6 h after CLP revealed that Fer-1 treatment reverses the expression of a core set of pro-inflammatory and chemokine pathway genes induced by CLP at early time point, suggesting Fer-1 either broadly and potently suppresses CLP induced “cytokine and chemokine storm” at early stage of disease onset, and/or reduced the cellular component associated with high proinflammatory cytokine and chemokine profile in the septic lung.

### 3.3 Fer-1 specifically reduces neutrophil infiltration into the septic lung

**Fig. 4:**
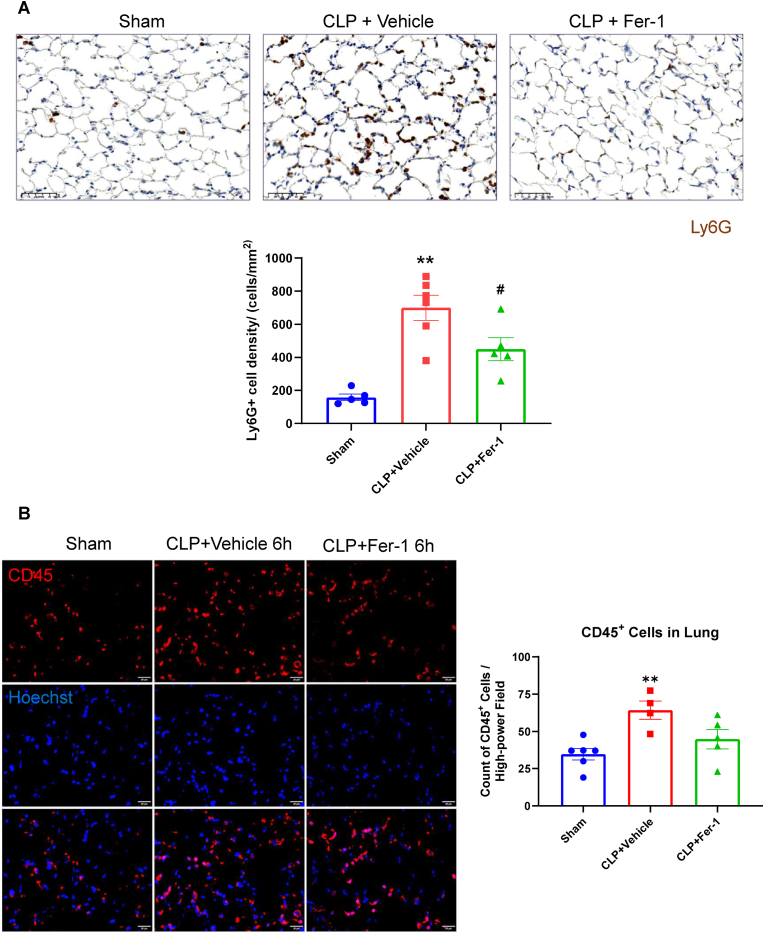
Fer-1 treatment significantly reduces Ly6G^+^ neutrophil infiltration in the septic lung. (A) Representative images and quantification of Ly6G^+^ cell density (neutrophils) in lung sections from Sham, CLP + Vehicle, and CLP + Fer-1 groups at 6 h post-CLP. Fer-1 treatment significantly reduced the density of Ly6G^+^ cells. N = 5, ∗∗*P* < 0.01 vs. Sham; #*P* < 0.05 vs. CLP + Vehicle analyzed by one-way ANOVA with Tukey's post-hoc test. (B) Representative immunofluorescence images and quantification of total leukocytes (CD45^+^, red) in lung sections from Sham, CLP + Vehicle, and CLP + Fer-1 groups at 6h. Nuclei are stained with Hoechst (blue). Data are presented as mean ± SEM. N = 4–6 mice per group, ∗∗P < 0.01 vs. Sham analyzed by one-way ANOVA with Tukey's post-hoc test. Data are presented as mean ± SEM.

Given that transcriptome analysis of Fer-1 treated septic lung revealed a reduced inflammation and chemokine signature, we sought to determine if Fer-1 treatment reduced immune cell infiltration, especially the fast-responding neutrophils. We stained the lung tissue for the specific neutrophil marker Ly6G and observed a dramatic and significant increase in Ly6G^+^ cell density in the CLP+Vehicle group (Fig. 4A). This massive neutrophil infiltration was significantly attenuated by Fer-1 treatment (Fig. 4A). A similar trend was observed for CD45^+^ cells (Fig. 4B). These data indicate that Fer-1 causes significant reduction of neutrophils infiltration in the septic lung tissue.

### 3.4 Fer-1 protects cell membrane integrity and reduces DAMP release via the NINJ1 pathway

Our *in vivo* data showed that Fer-1 significantly inhibits neutrophil lung infiltration, a process often triggered by DAMP released from injured cells [34] and amplified by the cytokines and chemokines released by the infiltrated neutrophils [35]. We therefore hypothesized that Fer-1 protects against lytic cell death and DAMP release. To investigate this, we employed a Hela-YFP quenching assay to monitor early membrane damage [36] and measured Propidium Iodide (PI) uptake and dsDNA release to quantify late-stage membrane rupture and DAMP release, respectively.

**Fig. 5:**
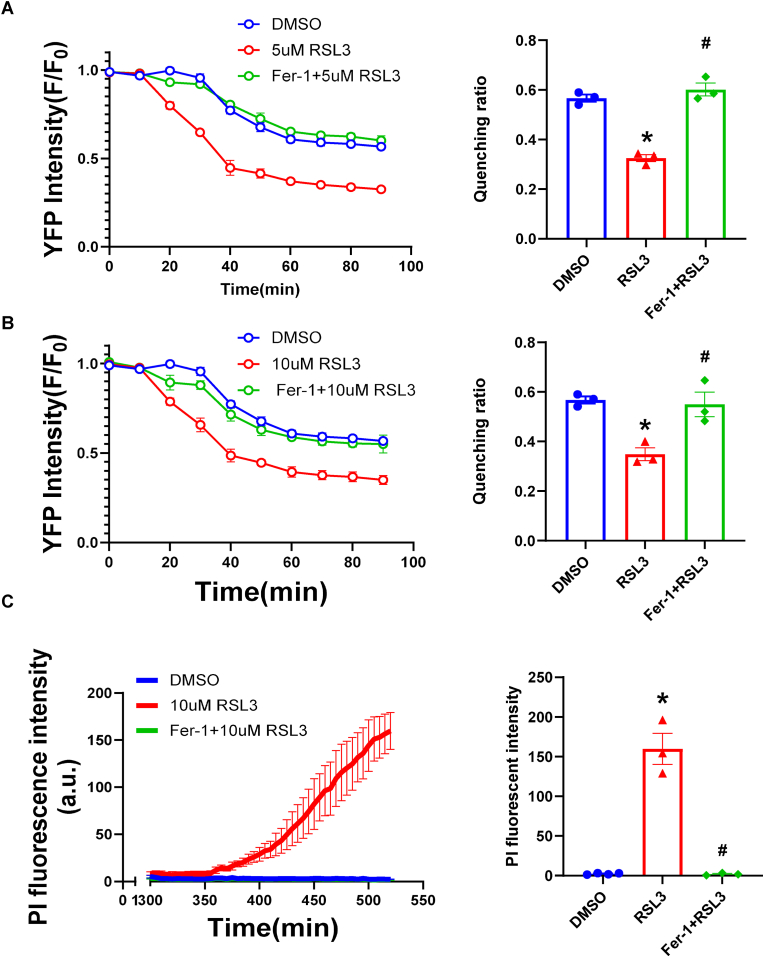
**Fer-1 protects cell membrane integrity against lipid-peroxidation-induced injury.** YFP quenching assay in HeLa-YFP cells treated with DMSO, RSL3 at 5 μM (A) or 10 μM (B), or RSL3 + 50 μM Fer-1. Time-course (left) and quantification of quenching ratio (right) at the last time point are shown, demonstrating Fer-1 rescues RSL3-induced quenching. (C) PI fluorescence intensity, indicating membrane rupture, in cells treated with DMSO, 10 μM RSL3, or 10 μM RSL3 + Fer-1. Time-course (left) and endpoint quantification (right) show Fer-1 blocks RSL3-induced cell lysis. Data are presented as mean ± SEM. ∗*P* < 0.05 vs. DMSO; #*P* < 0.05 vs. RSL3. N = 3 independent experiments, ∗*P* < 0.05 vs. DMSO; #*P* < 0.05 vs. RSL3 analyzed by one-way ANOVA with Tukey's post-hoc test.

First, we confirmed Fer-1's cytoprotective effects against ferroptotic stimuli. Treatment with the RSL3 (a covalent inhibitor of GPX4, at 5 μM and 10 μM) caused a rapid, time-dependent loss of YFP fluorescence in Hela cells, indicating membrane damage (Fig. 5A and B). Pre-treatment with 50 μM Fer-1 significantly rescued this YFP quenching at both RSL3 doses. Furthermore, massive membrane damage induced by RSL3 was shown by a sharp increase in PI fluorescence, which was almost completely blocked by Fer-1 pre-treatment (Fig. 5C).

**Fig. 6:**
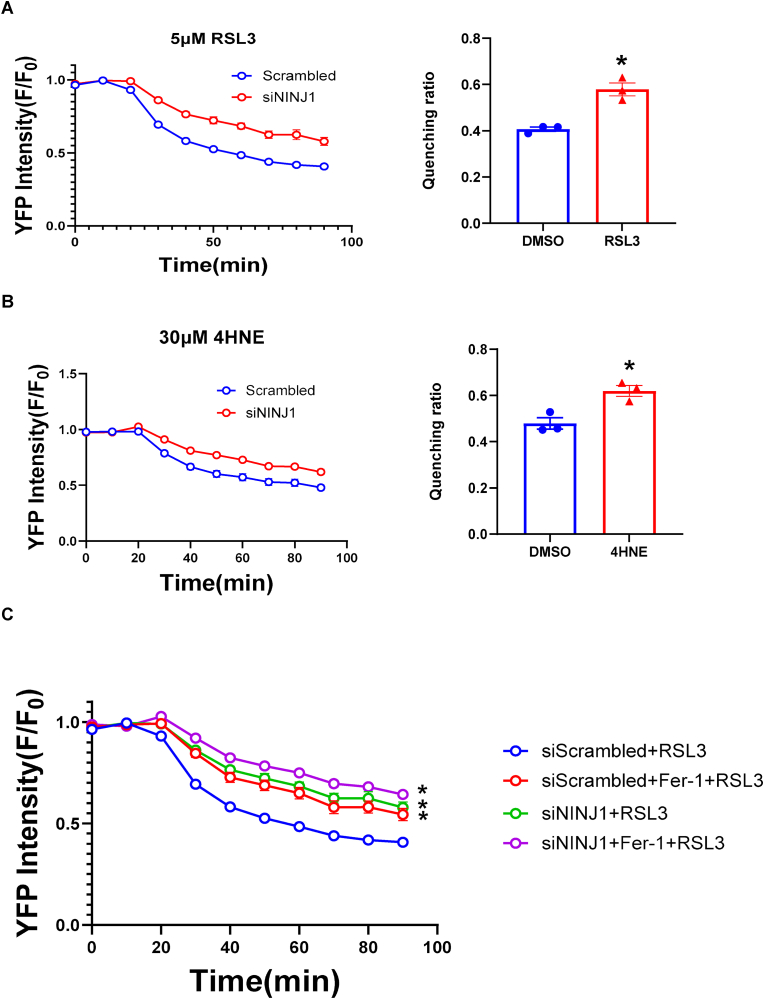
**The protective effect of Fer-1 against membrane damage is partially NINJ1-dependent.** YFP quenching assay in cells with Scrambled control or siNINJ1 knockdown, stimulated with 5 μM RSL3 (A) or 30 μM 4HNE (B). (C) Time-course and quantification of quenching ratio at the last time point of YFP quenching ratio in cells with indicated knockdowns and treatments. Fer-1 provides significant protection in siScrambled cells, but no additional protection in siNINJ1 cells. (D) Quantification of dsDNA (DAMP) release in supernatant from cells with indicated knockdowns and treatments. Both Fer-1 and siNINJ1 reduce dsDNA release, but their effects are not additive. N = 3 independent experiments, ∗*P* < 0.05 vs. siScrambled + RSL3 analyzed by one-way ANOVA with Tukey's post-hoc test. Data are presented as mean ± SEM. N = 3 independent experiments, ∗*P* < 0.05 vs. DMSO analyzed by unpaired, two-tailed Student's t-test in (A) and (B).

We next sought to determine if this membrane protection was mediated by NINJ1, a critical executioner of membrane damage and DAMP release during lytic cell death [30,33,36]. Knocking down NINJ1 significantly attenuated YFP quenching induced by both 5 μM RSL3 and 30 μM 4HNE (a toxic byproduct of ROS activity), compared to the control (Fig. 6A and B). It also significantly decreased PI uptake induced by both RSL3 and 4HNE (Supplemental Fig. 6). To test the link between Fer-1 and NINJ1, we performed an epistasis experiment. As observed before, both Fer-1 treatment and NINJ1 knockdown independently provided significant protection against RSL3-induced YFP quenching and PI uptake (Fig. 6C and. 7A and B). Importantly, adding Fer-1 to cells already lacking NINJ1 offered no additional protection compared to NINJ1 knockdown alone. This suggests that Fer-1 acting upstream to prevent the lipid peroxidation–dependent activation of NINJ1 pathway.

**Fig. 7:**
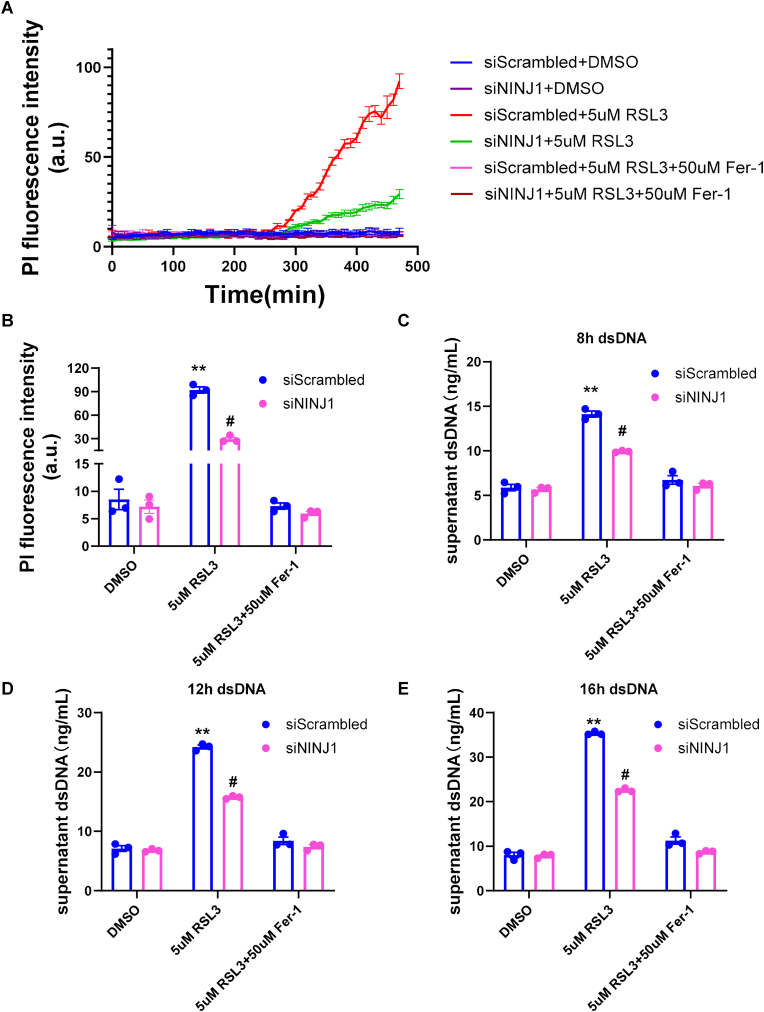
**Fer-1 prevents membrane rupture and dsDNA release via downstream NINJ1 pathway.** (A) A YFP quenching assay using HeLa-YFP cells treated with siScrambled or siNINJ1. Cells were stimulated with 5 μM RSL3 in the presence or absence of 50 μM Fer-1. Plasma membrane rupture was measured by the increase in Propidium Iodide (PI) fluorescence intensity over 500 min. (B) Quantification of the endpoint PI fluorescence intensity from panel A. (C–E) Measurement of dsDNA (ng/mL) in the cell supernatant at 8 h (C), 12 h (D), and 16 h (E) post-treatment. Cells were treated with siScrambled or siNINJ1 and stimulated with 5 μM RSL3, with or without 50 μM Fer-1, or a DMSO control. N = 3 independent experiments, ∗∗*P* < 0.01 vs. DMSO; #*P* < 0.05 vs. siScrambled analyzed by two-way ANOVA with Tukey's post-hoc test.

The protective effect of Fer-1 and the resulting lower DAMP release was further confirmed by measuring dsDNA (a large DAMP with high immunogenicity [37] in the supernatant. RSL3 caused a dramatic increase in dsDNA release, which was significantly reduced by either Fer-1 or siNINJ1 (Fig. 7C–E). Consistent with the YFP quenching data, the combination of Fer-1 and siNINJ1 provided no additive benefit.

**Fig. 8:**
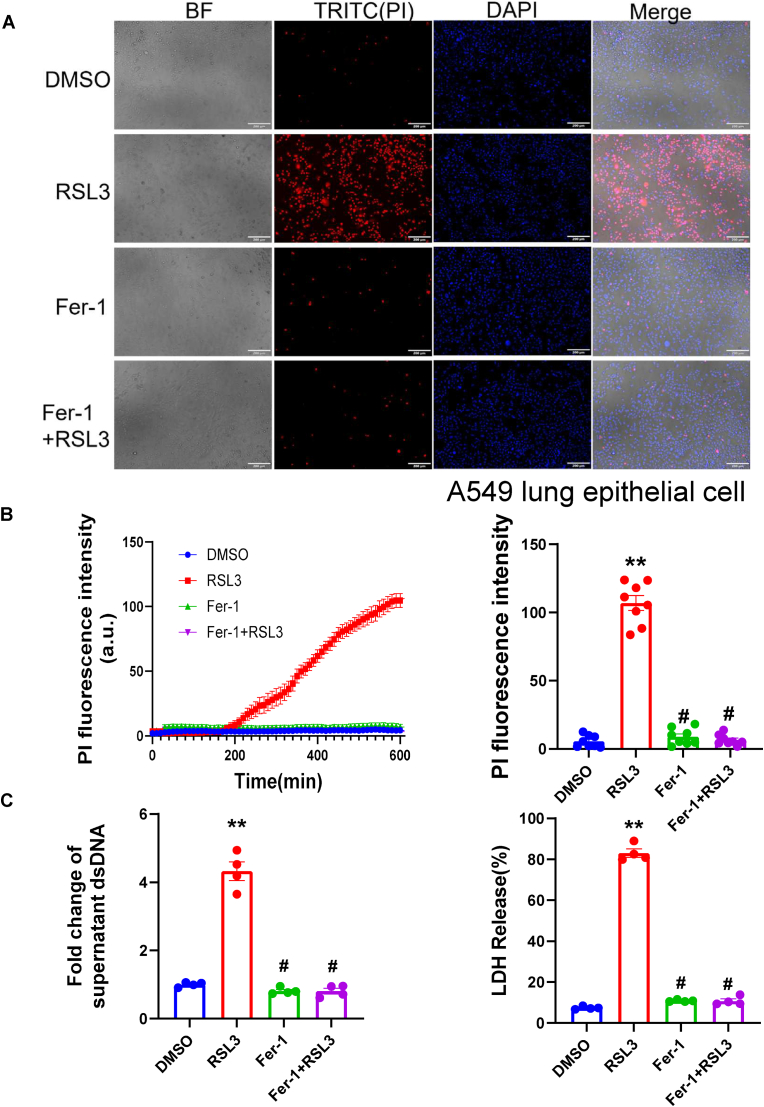
Ferrostatin-1 inhibits lipid-peroxidation-induced cell death and DAMP release in A549 lung epithelial cells. (A) Representative fluorescence images of A549 cells treated with DMSO (control), RSL3, Fer-1, or a combination of Fer-1 and RSL3. Cells were stained for Propidium Iodide (PI, red) to detect cell permeability/death and DAPI (blue) for nuclei. BF denotes Brightfield. (B) Real-time measurement of PI fluorescence intensity in A549 cells over 600 min (left panel) and quantification of endpoint PI intensity (right panel). (C) Quantification of DAMP release in the supernatant. The bar charts show the fold change of released dsDNA (left panel) and the percentage of Lactate Dehydrogenase (LDH) release (right panel) after treatment with DMSO, RSL3, Fer-1, or Fer-1+RSL3. Data are presented as mean ± SEM. (B) N = 8 independent experiments, ∗∗*P* < 0.01 vs. DMSO, #*P* < 0.05 vs. RSL3 analyzed by one-way ANOVA with Tukey's post-hoc test. (C) N = 3 independent experiments, ∗∗*P* < 0.01 vs. DMSO, #*P* < 0.05 vs. RSL3 analyzed by one-way ANOVA with Tukey's post-hoc test.

To investigate if the protective effects of Fer-1 also exist in lung derived cell lines, we tested A549 lung epithelial cells and lung endothelial cells. Treatment with RSL3 caused massive lytic cell death in lung epithelial cells, as shown by a dramatic increase in PI fluorescence, LDH release, and dsDNA release into the supernatant (Fig. 8A–C). Pre-treatment with Fer-1 almost completely blocked RSL3-induced lytic cell death and DAMP release. However, Fer-1 showed no effects of preventing lytic cell death induced DAMP release in lung endothelial cells (Supplemental Fig. 7). Taken together, these data strongly indicate that Fer-1 maintains cell membrane integrity and might reduce lung injury by inhibiting a lipid-oxidation-induced, NINJ1-mediated DAMP release from epithelial cells.

### 3.5 Fer-1 directly suppresses the neutrophil inflammatory response in vitro

**Fig. 9:**
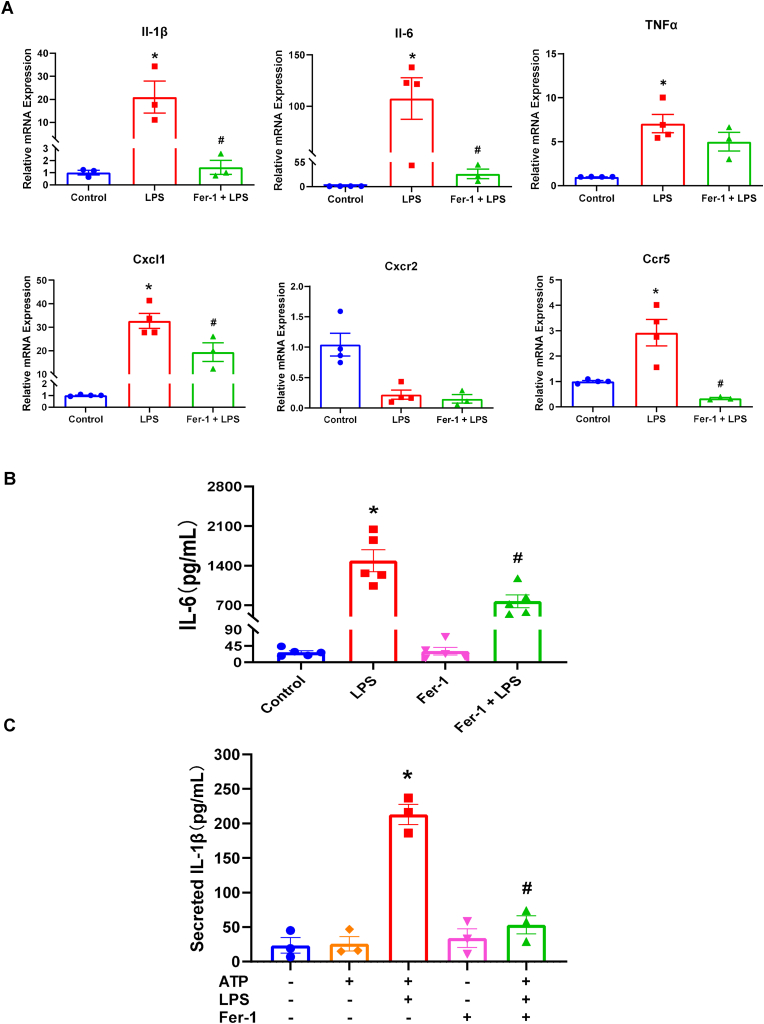
**Fer-1 directly suppresses LPS-induced cytokine production in neutrophils** (A) Relative mRNA expression of *IL-1β, IL-6, TNFα, Cxcl1, Cxcr2,* and *Ccr5* in primary neutrophils treated with Control, LPS, or Fer-1 + LPS. (B) Protein secretion of IL-6 (pg/mL) and secreted IL-1β (pg/mL) from neutrophils with indicated treatments (C) Relative mRNA expression of IL-1β and IL-6 in neutrophils treated with Control (Con), LPS, Fer-1, LPS + Fer-1, or LPS + Fer-1+Anisomycin (Aniso). Data are presented as mean ± SEM. N = 3–5 groups (each group includes neutrophils from two mice), ∗*P* < 0.05 vs. Control, #*P* < 0.05 vs. LPS analyzed by one-way ANOVA with Tukey's post-hoc test in (A); ∗*P* < 0.05 vs. Control, #*P* < 0.05 vs. LPS analyzed by two-way ANOVA with Tukey's post-hoc test in (B) and (C).

To determine Fer-1's mechanism of action on neutrophils, we isolated primary peritoneal neutrophils (identified by Ly6G^+^ staining, Supplemental Fig. 8A) and stimulated them with LPS in vitro. Fer-1's suppression of IL-1β was dose-dependent, with significant inhibition of both IL-1β and IL-6 mRNA observed at 50 μM of Fer-1 (Fig. 9A; Supplemental Fig. 8B). Consistent with our *in vivo* sepsis lung tissue data, Fer-1 significantly attenuated LPS induced expression of proinflammatory cytokines IL-1β and IL-6, but not TNFα, as well as the chemokine CXCL1 and chemokine receptor CCR5 (Fig. 9A). LPS stimulation in neutrophil also induced the ferroptosis-related gene *Ptgs2*, which was significantly inhibited by Fer-1 (Supplemental Fig. 8C). However, unlike the *in vivo* data, Fer-1 did not significantly suppress *Tnfa*, *Fth1* or *Slc7a11* expression in neutrophil (Fig. 9A; Supplemental Fig. 8C).

**Fig. 10:**
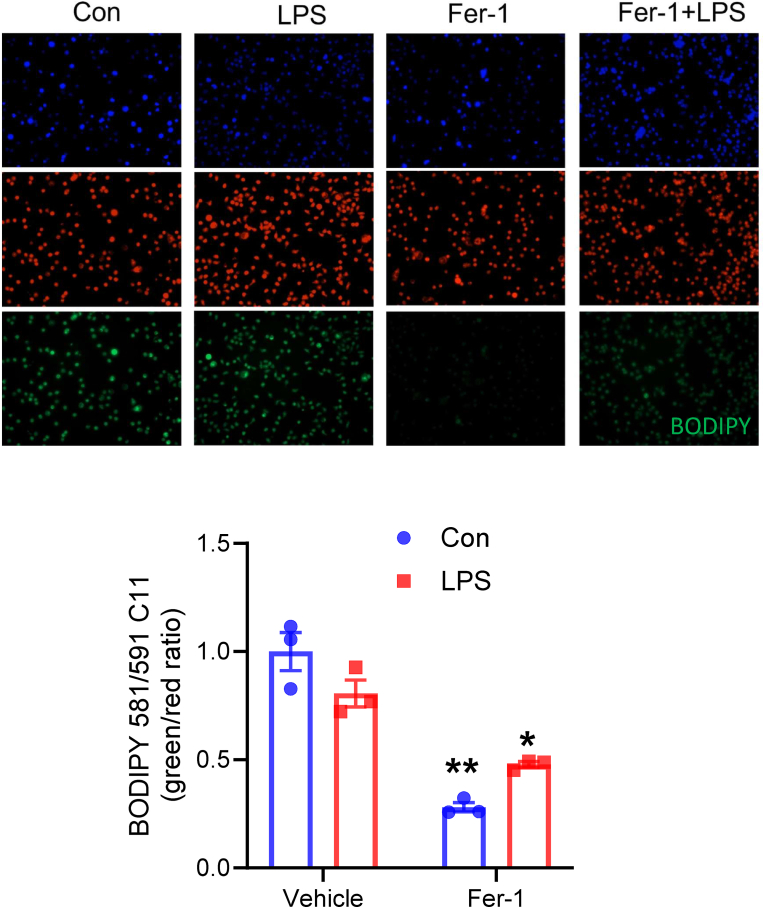
**Fer-1 acts as a potent radical-trapping antioxidant in neutrophils.** (Top) Representative fluorescence images of neutrophils stained with BODIPY 581/591 C11 (bottom, green = oxidized; middle, red = unoxidized) and Hoechst (top, blue = nuclei) under Control, LPS, Fer-1, or LPS + Fer-1 conditions. (Bottom) Quantification of the BODIPY 581/591 C11 green/red fluorescence ratio, indicating lipid peroxidation. Data are presented as mean ± SEM. N = 3 groups (each group includes neutrophils from two mice), ∗∗*P* < 0.01 vs. Vehicle, analyzed by two-way ANOVA with Tukey's post-hoc test.

To confirm that Fer-1 can inhibit the pro-inflammatory cytokine release from neutrophil, ELISA was used to detect the concentration of IL-6 and IL-1β protein in the culture medium of neutrophil. Fer-1 significantly blocked the protein secretion of both IL-6 and IL-1β from LPS-stimulated neutrophils (Fig. 9B and C). We next explored the mechanism of this suppression. We first confirmed Fer-1's role as a radical-trapping antioxidant using the lipid peroxidation probe BODIPY 581/591 C11. Fer-1 treatment alone significantly reduced basal levels of lipid peroxidation, confirming its potent antioxidant activity in neutrophils (Fig. 10). ROS has been shown to direct activate JNK/p38 pathway to induce inflammatory cytokine expression [38,39]. We co-treated cells with Anisomycin (Aniso), a known JNK/p38 pathway activator. Anisomycin co-treatment completely reversed the inhibitory effect of Fer-1 on both IL-1β and IL-6 mRNA expression (Supplemental Fig. 8D). This suggests that Fer-1's direct anti-inflammatory effect on neutrophils is mediated, at least in part, through the suppression of the JNK/p38 signaling pathway. Together, these data have shown that Fer-1 significantly suppresses key pro-inflammatory cytokine production from neutrophil via its potent radical-trapping antioxidant activity and inhibition of JNK/p38 pathway, suggesting Fer-1 might suppress the inflammation-amplifying signal from neutrophils and blocking the feed-forward loop which normally transforms a controlled defensive response into cytokine storm and massive neutrophil infiltration.

## 4 Discussion

Sepsis remains a leading cause of mortality, primarily through its progression to multi-organ failure, with ALI being a critical and early complication. Lipid-peroxidation–induced cell damage has been implicated in several forms of sepsis-induced acute organ injury including cardiac dysfunction [40], intestinal barrier breakdown [23], encephalopathy [41], thymic involution [42], and kidney injury [43] during sepsis. Fer-1, a lipid peroxidation inhibitor and radical-trapping antioxidant has consistently shown beneficial effects in survival and multiple organ dysfunction in sepsis at ∼24–48 h post-injury [18,23,44], when systemic inflammation, cytokine storm, and overt tissue injury are already established. However, the earliest pathological events (within hours) that shape the trajectory of sepsis-associated ALI including initial DAMP release, neutrophil priming, and early inflammatory gene activation have remained largely uncharacterized. In this study, we demonstrate that Fer-1 confers a previously unrecognized robust protection against sepsis-induced early lung injury in the CLP model as early as 6 h after CLP, a time point preceding mortality. This suggests that Fer-1's survival benefit starts at the early stage of the disease. Our data further demonstrated a dual mechanism for Fer-1's protective effects, suggesting it breaks the vicious cycle of inflammation that drives septic ALI by acting both upstream, to prevent DAMP release from lung epithelial cells, and downstream, to directly disarm the inflammatory response of infiltrating neutrophils.

Consistent with previous publications [25], our study demonstrates that pre-treatment with the potent radical-trapping antioxidant Fer-1 [17] significantly improves 48-h survival in a murine CLP-induced sepsis model. Importantly, Fer-1 also markedly preserved alveolar architecture and reduced histological lung injury as early as 6 h after CLP. This early attenuation of lung damage was accompanied by significantly reduced transcriptional expression of IL-1β, IL-6, and TNFα, suggesting that Fer-1 effectively blunts the initial inflammatory status in the lung. Our RNA-seq analysis revealed that CLP induces a rapid and massive transcriptional remodeling of the lung within 6 h, with >4000 genes altered. Remarkably, Fer-1 reversed over 1100 CLP-induced genes, including a core set of 185 genes enriched in inflammatory pathways such as IL-17, TNF, NF-κB, TLR, and chemokine signaling. These pathways are well-known drivers of neutrophil recruitment, activation, and inflammatory amplification. The transcriptional suppression of these pathways was reflected structurally by significantly reduced Ly6G + neutrophil infiltration into lung tissue, consistent with the central role of neutrophils in early ALI pathogenesis.

Because neutrophil infiltration in sepsis-associated ALI is strongly driven by membrane damage associated DAMP release, including dsDNA, histones, S100 proteins, and oxidized lipids [[45], [46], [47]], we investigated whether Fer-1 prevents DAMP release from cell membrane damage. Using a YFP-quenching membrane integrity assay [36], PI uptake, and dsDNA release, we demonstrate that Fer-1 robustly inhibits lipid-oxidation–induced membrane damage in vitro. We and others [30,31,33,36,48] have shown that NINJ1 is an executioner of plasma membrane damage and rupture during lytic cell death to mediate large DAMP release. *Ninj1* mRNA was significantly upregulated in septic lung tissue *in vivo*. *In vitro*, we found that NINJ1 knockdown partially phenocopied Fer-1's protective effect, preventing membrane damage and dsDNA release after RSL3-and 4HNE-induced lipid oxidation. Notably, epistasis analysis showed that Fer-1 and NINJ1 knockdown provided comparable levels of protection, and their combination was not additive. This genetic epistasis indicates that Fer-1 protects membrane integrity partially through a NINJ1-dependent downstream pathway, aligning with recent observations that lipid peroxidation serves as an upstream inducer for NINJ1-mediated membrane damage and rupture [32,33]. Fer-1 does not alter Ninj1 transcription but reduces NINJ1-dependent membrane rupture. Prior structural and functional studies demonstrate that NINJ1 oligomerization and membrane-destabilizing activity are influenced by lipid peroxidation–associated changes in membrane biophysics, including curvature stress and lipid-packing defects [33,49,50]. Ferroptosis-associated oxidized phospholipids such as 15-HpETE-PE and 12-HpETE-PE have been identified as important lipids involved in membrane instability [[51], [52], [53]]. This strongly implies that Fer-1's function as a radical-trapping antioxidant acts upstream of NINJ1, preventing the build-up of lipid peroxides that might function as an upstream signal for NINJ1 activation.

Moreover, Fer-1 suppressed dsDNA release from RSL3 treated lung epithelial cells but not from lung endothelial cells, suggesting that epithelial cells serve as a critical, lipid-oxidation-sensitive cell type that initiates the inflammatory cascade during septic ALI. As dsDNA is among the most potent large DAMPs initiating neutrophil chemotaxis and inflammatory amplification, these findings support a mechanistic model in which Fer-1 interrupts the early DAMP-driven recruitment loop that normally accelerates neutrophilic lung injury. Although direct comparative lipidomics for lung epithelial versus endothelial cells are not currently established, this differential sensitivity may stem from intrinsic redox-biological differences. Ferroptosis susceptibility is heavily influenced by the abundance of polyunsaturated phospholipids (PUFA-PLs) and the activity of lipid-remodeling enzymes like ACSL4 and LPCAT3 [54,55], as well as compartment-specific antioxidant defenses such as GPX4 activity and glutathione availability [56,57]. It is therefore plausible that the pulmonary epithelium possesses a lipid composition or metabolic profile that renders it more susceptible to ferroptotic oxidation than the endothelium, making it the primary beneficiary of Fer-1 protection in this context.

In addition to shaping the inflammatory environment, Fer-1 also acts directly on neutrophils. LPS-stimulated primary neutrophils treated with Fer-1 exhibited significant suppression of IL-1β and IL-6 mRNA and protein secretion, as well as reduced expression of CXCL1 and CCR5. Fer-1 lowered basal lipid peroxidation levels in neutrophils, consistent with its radical-trapping antioxidant activity. Importantly, activation of the JNK/p38 pathway with anisomycin fully reversed the anti-inflammatory effect of Fer-1 on IL-1β and IL-6 expression, suggesting that Fer-1 restrains neutrophil inflammatory output by reducing ROS-dependent activation of stress kinase pathways. Thus, Fer-1 not only reduces the number of neutrophils arriving at the lung but also might directly suppress the “amplification” signaling of the neutrophils that are present, effectively silencing the positive-feedback loop. This suppression of neutrophil activation must be viewed in the broader context of neutrophil biology in sepsis. Neutrophils in sepsis often display a phenotype known as “exhaustion,” characterized by both pathogenic inflammatory activity and a concurrent loss of proper function, such as impaired chemotaxis and bacterial clearance [58,59]. Given that NINJ1-mediated membrane rupture promotes chronic DAMP release and fuels inflammatory signaling, repeated exposure to these signals may contribute to neutrophil dysfunction over time. By limiting upstream DAMP release and directly suppressing JNK/p38-dependent cytokine production, Fer-1 may not only dampen acute inflammation but potentially mitigate the drive toward neutrophil exhaustion, though further studies in chronic sepsis models are needed to confirm this.

The progression of sepsis-induced ALI is driven by a devastating, self-amplifying loop: an initial insult causes lytic cell death, releasing DAMPs that trigger a massive chemokine response, which in turn recruit neutrophils [47]. These neutrophils, while attempting to clear the “threat,” release more toxic mediators and cytokines, amplifying the damage and recruiting more neutrophils [60,61]. Our data suggests Fer-1 breaks this cycle at its two most critical points by tackling the “upstream” trigger (DAMP release) and targeting the “downstream” inflammatory amplifier. Our study also provides a novel mechanistic link between lipid peroxidation, membrane damage and DAMP release via the membrane executioner protein NINJ1.

Finally, we must consider the translational relevance of these findings. Markers of lipid peroxidation, such as oxidized phospholipids, and NINJ1 expression are elevated in patients with sepsis and inflammatory conditions [62,63]. NINJ1 also has potential as a bioindicator of disease severity and prognosis in viral pneumonia and viral sepsis [64], suggesting the lipid-peroxidation–NINJ1–DAMP axis is relevant to human pathophysiology [64,65]. Regarding the timing of intervention, previous studies indicate that oxidized phospholipids accumulate rapidly within 1–3 h of septic insult [66,67]. Although our study utilized a pre-treatment paradigm to isolate early mechanistic events, the kinetics of lipid peroxidation suggest that early post-injury intervention might still effectively intercept NINJ1 activation and DAMP release. Future studies determining the precise therapeutic window for Fer-1 post-sepsis onset will be crucial for clinical translation.

In summary, our study provides a comprehensive, dual mechanism for Fer-1's protection in septic ALI. It acts as a potent radical-trapping antioxidant that 1) inhibits lipid-peroxidation-induced, NINJ1-mediated lytic death in lung epithelial cells, thereby preventing the “upstream” DAMP release that initiates the inflammatory cascade, and 2) directly suppresses the JNK/p38-mediated production of inflammatory cytokines in neutrophils, thereby breaking the “downstream” amplification loop. This positions Fer-1 and other inhibitors of the lipid peroxidation-NINJ1-DAMP axis as promising therapeutics for sepsis-induced ALI.

## 5 Conclusion

By defining Fer-1's effects within the first 6 h of CLP sepsis, our study identifies an early lipid-peroxidation-DAMP–NINJ1–neutrophil axis that drives sepsis-associated ALI. These findings expand the understanding of lipid-peroxidation associated tissue damage in sepsis beyond late-stage organ dysfunction and highlight the potential of targeting early lipid peroxidation and DAMP-mediated inflammatory loops as a therapeutic strategy. Since early intervention is critical for altering the trajectory of sepsis progression, these results provide compelling rationale for exploring Fer-1 and related radical-trapping antioxidants as modulators of early innate immune activation in sepsis-induced lung injury.

## Declaration of generative AI and AI-assisted technologies in the manuscript preparation process

During the preparation of this manuscript the authors used Google Germini 2.5-pro for word editing only to eliminate typos and grammatical errors. The authors then reviewed and edited the content as needed. The authors take full responsibility for the content of the manuscript.

## CRediT authorship contribution statement

**Fang Xiao:** Data curation, Formal analysis, Investigation, Methodology, Writing – original draft. **Donghua Li:** Data curation, Formal analysis, Investigation, Methodology, Writing – original draft. **Miao Yu:** Data curation, Formal analysis, Investigation, Methodology, Writing – original draft. **Yunfeng Zhu:** Data curation, Formal analysis, Investigation, Methodology, Writing – original draft. **Guorong Huang:** Data curation, Formal analysis, Investigation, Methodology, Writing – original draft. **Zhilei Huang:** Data curation, Formal analysis, Investigation, Methodology. **Yufang Wang:** Data curation, Formal analysis, Investigation, Methodology. **Jialin Li:** Investigation, Methodology. **Dongmei Zhong:** Investigation, Methodology. **Huan Ma:** Investigation. **Kunyu Liao:** Investigation, Methodology. **Yongshan Liu:** Investigation. **Yalin Zhang:** Investigation. **Xiangdong Guan:** Supervision. **Changjie Cai:** Resources. **Jing Tang:** Resources. **Tianqin Peng:** Conceptualization, Supervision, Writing – review & editing. **Fu-Li Xiang:** Conceptualization, Funding acquisition, Project administration, Supervision, Writing – original draft, Writing – review & editing. **Jie Xu:** Conceptualization, Funding acquisition, Supervision, Writing – original draft, Writing – review & editing.

## Declaration of competing interest

The authors declare that they have no known competing financial interests or personal relationships that could have appeared to influence the work reported in this paper.

## Data Availability

Data will be made available on request.
