# Supplementary material for: Ferrostatin-1 protects against early sepsis-induced acute lung injury by suppressing lipid peroxidation–driven NINJ1-mediated DAMP release and neutrophil activation
Source: Redox Biol. 2026 Jan 6;90:104004. doi: 10.1016/j.redox.2026.104004 (PMC12816856; doi:10.1016/j.redox.2026.104004)
Supplement: Multimedia component 1 [file mmc1.docx]

**Supplemental Methods**

**Animals**

All animal experiments were carried out in accordance with procedures approved by the Institutional Animal Care and Use Committee of Sun Yat-sen University (SYSU-IACUC-2024-002534). Male C57BL/6J mice of 8~12 weeks old were used in this research and purchased from Guangzhou Ruige Biological Technology Co., LTD. Before experiment, animals were housed under specific pathogen-free conditions in a 12 h light/dark cycle and temperature-controlled room (19~25 ℃) with free access to food and water.

**Cecal ligation and puncture model and Fer-1 treatment *in vivo***

As was described previously, 2 h before CLP-induced sepsis, mice of the CLP + Fer-1 group were i.p. injected with Fer-1 (Selleck Chemicals, S7243, 5 mg/kg), and CLP group were i.p. injected with vehicle for Fer-1. Mice were anesthetized with inhalation of 2% isoflurane and 1 L/min O_2_ under sterile conditions. The peritoneal cavity was cut open, the cecum was ligated at 1 cm from the distal end and the distal cecum was punctured for a single hole with 16G needle to induce sepsis. A small amount of fecal was squeezed out of the hole of the cecum before the cecum was returned to the peritoneal cavity. The incision was sutured with 5-0 silk suture. Subsequently, fluid resuscitation was performed by subcutaneous injection of 1 mL of 0.9% sterile saline. Sham-operated mice underwent the same surgical procedures, including laparotomy, but without cecal ligation or puncture.[1].

**Echocardiography**

Cardiac function was performed 6 h post CLP surgery using a small animal ultrasound system equipped with a 40-MHz transducer (Vevo 3100, Fujifilm Visualsonics Inc.)[2]. Mice were anesthetized with inhalation of 1~2% isoflurane and 0.5~1 L/min O_2_ in a supine position. LV parameters including ejection fraction percentage (EF), fraction shortening (FS), left ventricular internal diameter (LVID), left ventricular wall thickness and speckle tracking based overall longitudinal strain (GLS) were measured using M and B mode of the LV long-axis view. E/A of mitral valve was measured using pulsed wave Doppler of the apex four-cavity view. EF, FS, LVID, LV wall thickness and E/A were calculated as an average over five consecutive cardiac cycle excluding respiratory peak. GLS was calculated by tracing the movement of endocardium over two consecutive cardiac cycle[3].

**Histological staining**

The lung tissues were fixed in 4% paraformaldehyde and subsequently embedded in paraffin. 5 μm sections were deparaffinized and rehydrated through a gradient ethanol series before staining. Sections were subjected to antigen retrieval with citrate buffer (pH 6.0) under high pressure heating or microwave heating before blockage. Then, sections were incubated with following primary antibodies overnight at RT: anti-CD45 rabbit mAb (1:100, CST, 70257S), anti-CD68 rabbit mAb (1:200, CST, 97778S), anti-LY6G rabbit pAb (1:100, Servicebio, GB11229) and anti-MPO rabbit pAb (Immunoway, YT5351). For immunofluorescence (IF), sections were subsequently incubated with anti-rabbit IgG (H+L) Alexa Fluor 594 (1:500, CST, 8889S) for 1 h and nuclei were counterstained with Hoechst 33342 for 10 minutes at RT. For Immunohistochemistry (IHC), sections were treated with 3% H_2_O_2_ to inactivate endogenous peroxidase followed by incubation with HRP conjugated goat anti-rabbit IgG (1:400, Servicebio, GB23303) at RT for 45 minutes. HRP color was developed by 3, 3'-diaminobenzidine (DAB, Servicebio, G1212) and nuclei were counterstained by hematoxylin. IF images were obtained with Olympus Intelligent Microscope Model BX63 and IHC images were captured with Kfbio Digital Pathology Slide Scanner KF-PRO-020[1].

**Lung injury score analysis**

Determination of the lung injury was performed by an independent examiner blind to the group assignment. Briefly, five regions from each specimen were assesed from 0 to 4 according to the degree of severity (0: not observed, 1: mild, 2: moderate, 3: strong, 4: intense) for congestion, interstitial thickness/infiltration, edema and hemorrhage. The sum of different parameters was calculated as total scores of lung injury[4, 5].

**Serum preparation and iron assay**

As was described before, the serum was collected from mice 6 h after CLP surgery[1]. The whole blood was collected and left still at RT for 30 minutes. Then samples were centrifuged at 12000 ×g at 4 °C for 10 minutes and the supernatant serum was transferred into new tubes and stored at -80 °C. The concentration of ferric (Fe^3+^) and ferrous (Fe^2+^) cation in serum were measured by Iron Assay kit (Abcam, ab83366) in accordance with the manufacturer’s instruction. Briefly, the serum was incubated with iron probe at 37 °C for 1 h avoided from light followed by determination of the OD593 of samples (Varioskan LUX, Thermo Scientific). The concentration of ferrous (Fe^2+^) cation and total iron (Fe^2+^ + Fe^3+^) cation were absolutely quantified by standard curve.

**Cell Culture and treatment**

HeLa (ATCC Cat#CCL-2, RRID: CVCL_0030), HEK293T (ATCC Cat#CRL-3216, RRID: CVCL_0063) and A549 (ATCC Cat# CCL-185, RRID: CVCL_0023) cells were obtained from American Type Culture Collection (ATCC). HeLa YFP cells were isolated as single-cell-derived clones through flow cytometry sorting after transfection with the halide-sensitive YFP (with H1480 and l152L mutations) plasmid. Mouse pulmonary endothelial cells (CP-M001) were obtained from Procell. All of the cell lines are frequently checked by morphological features and functionalities, but have not been subjected to authentication by short-tandem-repeat profiling. All of the cell lines have tested negative for the presence of mycoplasma by a detection kit (Vazyme, D101-02). The HeLa YFP and HEK293T cells were grown cultured in Dulbecco's modified Eagle's medium (DMEM) (Gibco, C11330500BT) containing 4.5 mg ml^-1^ glucose, supplemented with 10% FBS (ExCellBio, FND500) and 1% penicillin-streptomycin (Gibco,15140122). A549 cells were grown cultured in RPMl-1640 medium (Gibco, C22400500BT), supplemented with 10% FBS (ExCellBio, FND500) and 1% penicillin-streptomycin (Gibco,15140122). Mouse pulmonary endothelial cells were grown cultured in Endothelial Cell Medium (ECM) (ScienCell, 1001), supplemented with 5% FBS (ScienCell, 0025), 1% endothelial cell growth supplement (ScienCell, 1052) and 1% penicillin-streptomycin (ScienCell, 0503). All cells were kept in a humidified incubator set at 37 °C with 5% CO_2_ with medium refreshment carried out every other day. RSL3 (Selleck, 1219810-16-8), Ferrostatin-1 (Selleck, S7243), 4-HNE (Selleck, S9793), Nigericin (Selleck, S6653), TNFa (R&D, 410-MT-010), LPS (Meilunbio, MB5198), Anisomycin (Selleck, S7409) were used to treat different cell lines. The concentration and time were described in the figure legends. Cells were collected for RNA isolation or immunoblot and supernatant was collected for measurement of released cytokines, LDH and dsDNA

**Plasmid and siRNA transfection**

siRNA transfection was performed using Lipofectamine RNAiMAX (Invitrogen, 13778-150) according to the manufacturer’s instructions. Hela YFP Cells or A549 cells were seeded onto 12-well plates and transfected at approximately 70–80% confluency. siRNA was added to the culture system at a concentration of 20 nM. After 48 hours of transfection, cells were subjected to the indicated treatments. The sequences of siRNA (GenePharma, A01001) targeting human NINJ1 were as follows:NINJ1-homo-318 5′-GGGUGCUGCUCAUCUUCCU-3′;NINJ1-homo-386 5′-GUGGUAGUCAACAUCUUCA-3′;NINJ1-homo-1046 5′-GGCUCUGGUCUUAAGUGCC-3′.

FuGENE HD Transfection Reagent (Promega, E2311) was used to do transient plasmid transfection according to the manufacturer’s instructions. HEK293T cells were seeded onto 24-well plates and transfected with CMV-hNINJ1-IRES-GFP which modified from CMV-hNINJ1-IRES-mCherry[7] plasmid at 200 ng per well. After 24 hours, a series of doses of Ferrostatin-1 was added for 1 hour, then TNFa (10 ng/ml) was added for 4 hours or nigericin (10 ug/ml) for 2 hours respectively. Cells was collected and extracted for proteins.

**Neutrophil isolation and treatment**

Mice were i.p. injected with 1 mL 3% thioglycollate broth medium (Solarbio, LA8740). After 4~6 hours, mice were euthanized and neutrophils were collected by peritoneal lavage with 10 mL ice-cold RPMl-1640 medium supplemented with 1% FBS and 1% PS. Neutrophils were seeded and treated with 50 μM Ferrostatin-1 (Selleck, S7243) for 1 hour followed by 50 ng/mL Lipopolysaccharide originated from *E. coli* 055: B5 (Meilunbio, MB5198) for 4 hours. Cells were collected for RNA isolation or immunoblot and supernatant was collected for measurement of released cytokines. For secretion of IL-1β, 5 mM ATP was added 1 hour before termination. 100ng/mL Anisomycin (Selleck, S7409) was added 30 minutes before adding LPS.

**Induction of NETosis and Immunofluorescence Staining**

Cell viability was assessed by fluorescent staining of neutrophils using the nucleic acid dyes Hoechst 33342 (Catalog No.: H3570, Thermo Fisher Scientific, USA) and SYTOX™ Green nucleic acid stain (Catalog No.: S7020, Thermo Fisher Scientific, USA). After treatment, the cell culture medium was removed and replaced with a working solution containing a mixture of Hoechst 33342 and SYTOX Green, achieving final concentrations of 5 µg/mL and 50 nM, respectively. The cells were incubated in the dark for 20 minutes under light-protected conditions. Following incubation. Imaging was performed using an inverted fluorescence microscope (Model: IX83, Olympus, Japan) equipped with a chilled CCD camera (Model: DP80, Olympus, Japan). Images were acquired under appropriate fluorescence channels for subsequent analysis.

**LY6G staining**

Neutrophils were stained with anti-LY6G rat antibodies (1:100, Biolegend, 127601) and then with anti-Rat IgG (H+L) Alexa Fluor™ 594 (1:500, Invitrogen, A-21209)[8]. Cells were imaged with an inverted fluorescence microscope (Olympus, BX63F). Image analysis was performed in OlyVia and ImageJ software.

**BODIPY-C11 staining**

The probe kit of BODIPY-C11(HY-D1301, MCE) was used to assess lipid peroxidation, following the manufacturer’s instructions. Briefly, the cells were washed with Hank's Balanced Salt Solution (HBSS) (Solarbio, H1025-500ml) three times. 3uM BODIPY-C11 was added to the cells for 15 minutes at room temperature. After washing with HBSS three times, the cells were stained with Hoechst 33342 for 10 minutes at room temperature. Cells were imaged with an inverted fluorescence microscope (Olympus, BX63F). Image analysis was performed in OlyVia and ImageJ software.

**RNA isolation and real-time quantitative PCR**

Total RNA from cells and lungs of sham, CLP, CLP + Fer-1 group was extracted with RNAiso Plus (TAKARA, 9109) in accordance with the manufacturer’s instruction. RNA was reverse-transcribed using reverse transcription kit with random primers (TAKARA, RR047A) and conducted with quantitative real-time PCR kit (TAKARA, RR820) for 40 cycles on an ABI QuantStudio 5 System. The mRNA expression of target genes was assessed, with 18S serving as the internal reference gene. Relative expression levels were calculated using the 2^-ΔΔCt^ method. The oligonucleotide primers used in QRT-PCR were listed as follow:

| **Gene** | **Primer (5'→3')** | **Sequence** |
| --- | --- | --- |
| 18s | Forward primer | GCCGCTAGAGGTGAAATTCTT |
|  | Reverse primer | CGTCTTCGAACCTCCGACT |
| mCcl2 | Forward primer | GCTACAAGAGGATCACCAGCAG |
|  | Reverse primer | GTCTGGACCCATTCCTTCTTGG |
| mCcl3 | Forward primer | ACTGCCTGCTGCTTCTCCTACA |
|  | Reverse primer | ATGACACCTGGCTGGGAGCAAA |
| mCcl4 | Forward primer | TTCCTGCTGTTTCTCTTACACCT |
|  | Reverse primer | CTGTCTGCCTCTTTTGGTCAG |
| mCcl5 | Forward primer | CCTGCTGCTTTGCCTACCTCTC |
|  | Reverse primer | ACACACTTGGCGGTTCCTTCGA |
| mCcl7 | Forward primer | CAGAAGGATCACCAGTAGTCGG |
|  | Reverse primer | ATAGCCTCCTCGACCCACTTCT |
| mCcl11 | Forward primer | TCCATCCCAACTTCCTGCTGCT |
|  | Reverse primer | CTCTTTGCCCAACCTGGTCTTG |
| mCcl17 | Forward primer | CGAGAGTGCTGCCTGGATTACT |
|  | Reverse primer | GGTCTGCACAGATGAGCTTGCC |
| mCcl22 | Forward primer | GTGGAAGACAGTATCTGCTGCC |
|  | Reverse primer | AGGCTTGCGGCAGGATTTTGAG |
| mCcr1 | Forward primer | CTCATGCAGCATAGGAGGCTT |
|  | Reverse primer | ACATGGCATCACCAAAAATCCA |
| mCcr2 | Forward primer | GCTGTGTTTGCCTCTCTACCAG |
|  | Reverse primer | CAAGTAGAGGCAGGATCAGGCT |
| mCcr5 | Forward primer | TTTTCAAGGGTCAGTTCCGAC |
|  | Reverse primer | GGAAGACCATCATGTTACCCAC |
| mCxcl1 | Forward primer | TCCAGAGCTTGAAGGTGTTGCC |
|  | Reverse primer | AACCAAGGGAGCTTCAGGGTCA |
| mCxcl2 | Forward primer | CATCCAGAGCTTGAGTGTGACG |
|  | Reverse primer | GGCTTCAGGGTCAAGGCAAACT |
| mCxcl3 | Forward primer | TGAGACCATCCAGAGCTTGACG |
|  | Reverse primer | CCTTGGGGGTTGAGGCAAACTT |
| mCxcl5 | Forward primer | CCGCTGGCATTTCTGTTGCTGT |
|  | Reverse primer | CAGGGATCACCTCCAAATTAGCG |
| mCxcl15 | Forward primer | GGTGATATTCGAGACCATTTACTG |
|  | Reverse primer | GCCAACAGTAGCCTTCACCCAT |
| mCxcr1 | Forward primer | CCATTCCGTTCTGGTACAGTCTG |
|  | Reverse primer | GTAGCAGACCAGCATAGTGAGC |
| mCxcr2 | Forward primer | CTCTATTCTGCCAGATGCTGTCC |
|  | Reverse primer | ACAAGGCTCAGCAGAGTCACCA |
| mFth1 | Forward primer | GCCGAGAAACTGATGAAGCTGC |
|  | Reverse primer | GCACACTCCATTGCATTCAGCC |
| mGpx4 | Forward primer | CCTCTGCTGCAAGAGCCTCCC |
|  | Reverse primer | CTTATCCAGGCAGACCATGTGC |
| mHamp | Forward primer | CAGCACCACCTATCTCCATCAAC |
|  | Reverse primer | CAGATGGGGAAGTTGGTGTCTC |
| mHmox1 | Forward primer | CACTCTGGAGATGACACCTGAG |
|  | Reverse primer | GTGTTCCTCTGTCAGCATCACC |
| mIl1b | Forward primer | AGGCTCCGAGATGAACAACA |
|  | Reverse primer | TTGTCGTTGCTTGGTTCTCC |
| mIl-6 | Forward primer | CAACGATGATGCACTTGCAGA |
|  | Reverse primer | GGTACTCCAGAAGACCAGAGG |
| mTnfα | Forward primer | GATCGGTCCCCAAAGGGATG |
|  | Reverse primer | TGAGGGTCTGGGCCATAGAA |
| mNinj1 | Forward primer | GTGGTCCTCATCTCTATCTCCC |
|  | Reverse primer | CGACGATGATGAAAACCAGTCCC |
| hNINJ1 | Forward primer | TCTCCATCTCCCTTGTGCTGCA |
|  | Reverse primer | CTACCACGATGATGAACACCAGG |
| mPtgs2 | Forward primer | GCGACATACTCAAGCAGGAGCA |
|  | Reverse primer | AGTGGTAACCGCTCAGGTGTTG |
| mSlc7a11 | Forward primer | CTTTGTTGCCCTCTCCTGCTTC |
|  | Reverse primer | CAGAGGAGTGTGCTTGTGGACA |
| mSrgn | Forward primer | TGCGAACTGCATCGAGGAGAAG |
|  | Reverse primer | CCCGAACCTGACCCATAGTCAT |

**Protein isolation and immunoblotting**

For native page, sample preparation was performed according to the manufacturer’s recommendations. In brief, the cells were washed once with PBS (Gibco, C10010500BT), and then 1× sample buffer containing 1% digitonin was added (Thermo Fisher Scientific, BN2008). The cells were scraped off by pipette tips and were transferred into tubes. The samples were centrifuged at 20,000g at 4 °C for 30 minutes and the supernatants were collected. The protein concentration was determined using a BCA protein assay kit (Beyotime, P0010). The protein was separated by 3–12% NativePAGE Bis-Tris Mini Protein Gels (Thermo Fisher Scientific, BN1002BOX) and transferred to PVDF membranes. the membrane was probed with primary antibodies anti-human NINJ1 antibody (1 µg ml^-1^, R&D, AF5105) and anti-GAPDH antibody (Proteintech, 60004-1-Ig). After incubation with HRP-conjugated anti-sheep secondary antibodies (abclonal, AS023, 1:5,000) or anti-mouse secondary antibodies (Proteintech, SA00001-2), immunoblots were visualized by using FDbio-Dura ECL Kit (Fdbio science, FD8020) and Amersham Image Quant800 (Cytiva).

**ELISA**

Cytokines (IL-1β, IL-6 and TNF-α) in cell supernatant were measured with Elisa kits (Neobioscience, EMC001b.96, EMC004.96, EMC102a(H).96) followed by manufacturer’s instructions. The sample absorbance at 450nm was measured by a microplate reader (ThermoFisher Scientific, Varioskan LUX).

**YFP quenching**

The YFP quenching assay was performed as previously described [7]. HeLa-YFP cells were seeded onto black clear-bottom 384-well plates and used at 70–80% confluency. Cells were washed and maintained in wash solution (140 mM NaCl, 5 mM KCl, 1 mM CaCl₂, and 20 mM HEPES; pH adjusted to 7.4 with NaOH). RSL3 or 4-HNE was added before time-lapse imaging. Fer-1 was added 1h before adding other stimuli. The cells were stained with 1 µg/ml propidium iodide (PI, Sigma, P4170-10MG). A high-iodide solution (120 mM NaCl, 20 mM NaI, 5 mM KCl, 1 mM CaCl₂, and 20 mM HEPES; pH 7.4) was applied during imaging to induce YFP quenching. YFP and PI fluorescence were recorded every 10 minutes using FITC and TRITC channels, respectively. YFP and PI fluorescence intensity was normalized to baseline values, and DMSO-treated cells were used as controls.

**LDH release assay**

LDH in cell culture supernatant were analyzed using the CytoTox 96Non-Radioactive Cytotoxicity Assay (Promega, G1780). The sample absorbance at 490nm was measured by a microplate reader (ThermoFisher Scientific, Varioskan LUX). LDH release was normalized to the untreated and 100% lysis control. LDH release was calculated as follows: LDH release (%) = (LDH _sample_-LDH _negative control_)/ (LDH _full lysis_-LDH _negative control_)

**dsDNA release assay**

The concentration of dsDNA in the cell culture supernatant was quantified using the PicoGreen dsDNA Quantification Kit (Solarbio, P9740). The fluorescence intensity was recorded using a microplate reader (ThermoFisher Scientific,Varioskan LUX; excitation,488 nm; emission 520 nm). The dsDNA concentration was determined by comparing the fluorescence values to a standard curve generated by dsDNA samples supplied with the kit.

**RNASeq Analysis**

Total RNA was extracted from lung tissues collected 6 hours after surgery from sham, CLP, and CLP + Fer-1 groups (n=2–3 mice per group, aged 8–12 weeks). RNA-seq was performed to obtain transcriptomic profiles. Genes with low expression (FPKM≤1 and read counts≤10) were excluded. Differentially expressed genes (DEGs) were identified using thresholds of p<0.05 and |log₂(fold change)|>1. Data processing and visualization were carried out using R software (version 4.5.1). A heatmap of the top 29 DEGs (FPKM≥30 and |log₂(fold change)|≥2) was generated with the “pheatmap” package. Gene Ontology (GO) and Kyoto Encyclopedia of Genes and Genomes (KEGG) pathway enrichment analyses were performed using the “clusterProfiler” package in R[9]. Transcription factor to target analyses were performed using Metascape.org (version3.5.20250701)[10].

**Statistical analysis**

All quantitative data are presented as mean ± SEM. Statistical analyses were performed using GraphPad Prism software (v9.0, GraphPad Software). For comparisons between two groups with normally distributed data, an unpaired, two-tailed Student’s t-test was used. For comparisons between more than two groups with normally distributed data, a one-way or two-way Analysis of Variance (ANOVA) was performed, followed by Tukey’s multiple comparisons post-hoc test. For data that were not normally distributed, the non-parametric Mann-Whitney U test (for two groups) or the Kruskal-Wallis test with Dunn's multiple comparisons test (for multiple groups) was used. Survival curves were generated using the Kaplan-Meier method and compared using the Log-rank (Mantel-Cox) test. A P-value < 0.05 was considered statistically significant. The specific tests used for each experiment are detailed in the figure legends.

**References:**

1. Li, J., et al., *Ferrostatin-1 improves acute sepsis-induced cardiomyopathy via inhibiting neutrophil infiltration through impaired chemokine axis.* Frontiers in Cell and Developmental Biology, 2024. **12**.

2. Kim, D., B. Langmead, and S.L. Salzberg, *HISAT: a fast spliced aligner with low memory requirements.* Nature Methods, 2015. **12**(4): p. 357-360.

3. Love, M.I., W. Huber, and S. Anders, *Moderated estimation of fold change and dispersion for RNA-seq data with DESeq2.* Genome Biology, 2014. **15**(12).

4. Xiao Lu, L.Z., Yong-Hua Xu, *Low molecular weight heparin prevents CLP-induced acute lung injury in rats by anti-inflammatory coagulation.* Bosn J Basic Med Sci, 2013(13): p. 50-56.

5. Störmann, P., et al., *Early Local Inhibition of Club Cell Protein 16 Following Chest Trauma Reduces Late Sepsis-Induced Acute Lung Injury.* Journal of Clinical Medicine, 2019. **8**(6).

6. Li, Z., et al., *Recurrent GNAQ mutation encoding T96S in natural killer/T cell lymphoma.* Nature Communications, 2019. **10**(1).

7. Zhu, Y., et al., *NINJ1 regulates plasma membrane fragility under mechanical strain.* Nature, 2025. **644**(8078): p. 1088-1096.

8. Guma, M., et al., *Caspase 1–independent activation of interleukin‐1β in neutrophil‐predominant inflammation.* Arthritis & Rheumatism, 2009. **60**(12): p. 3642-3650.

9. Wu, T., et al., *clusterProfiler 4.0: A universal enrichment tool for interpreting omics data.* The Innovation, 2021. **2**(3).

10. Zhou, Y., et al., *Metascape provides a biologist-oriented resource for the analysis of systems-level datasets.* Nature Communications, 2019. **10**(1).
