# Supplementary material for: Ferrostatin-1 protects against early sepsis-induced acute lung injury by suppressing lipid peroxidation–driven NINJ1-mediated DAMP release and neutrophil activation
Source: Redox Biol. 2026 Jan 6;90:104004. doi: 10.1016/j.redox.2026.104004 (PMC12816856; doi:10.1016/j.redox.2026.104004)
Supplement: Multimedia component 2 [file mmc2.pdf]

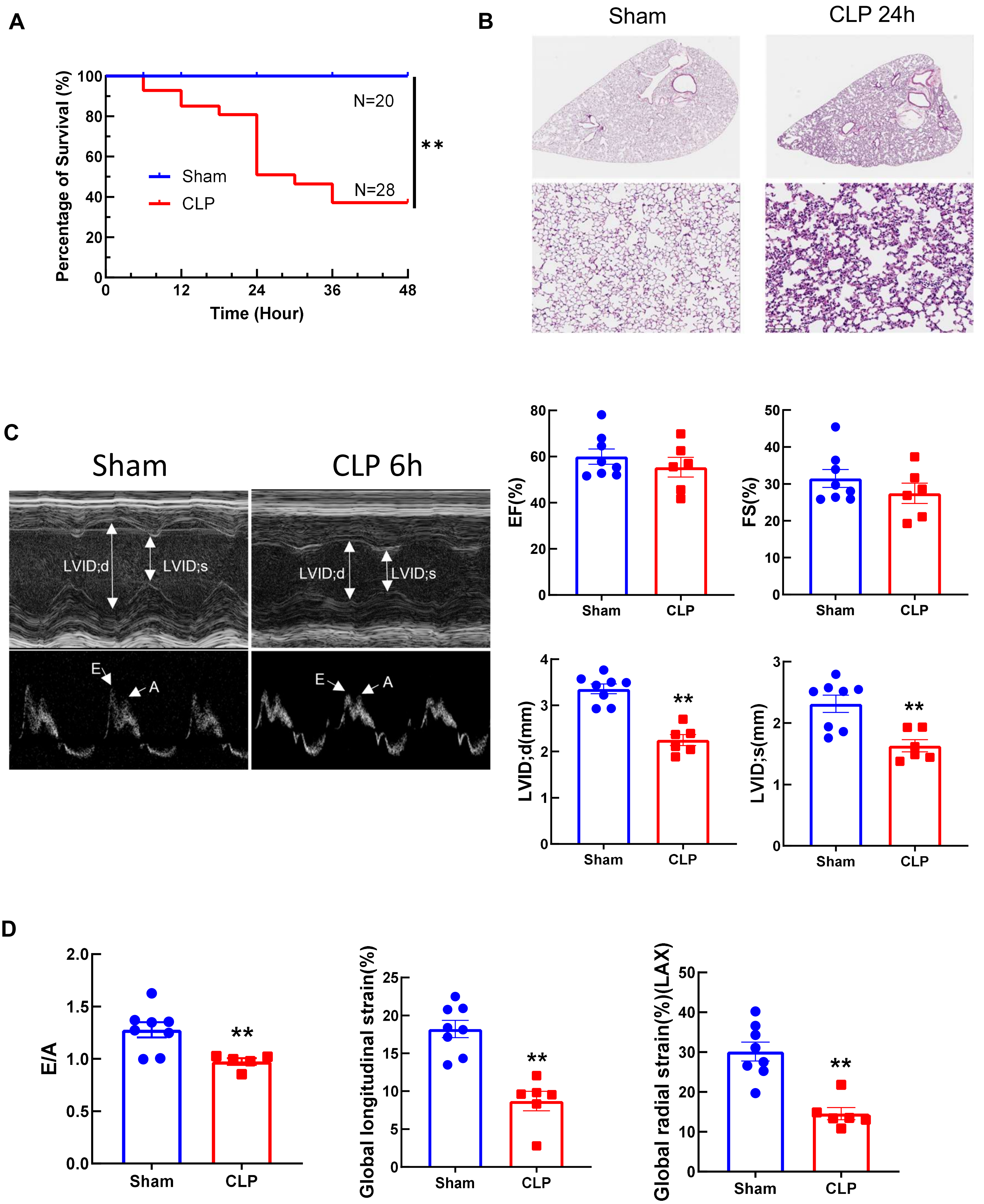

Supplemental Figure 1

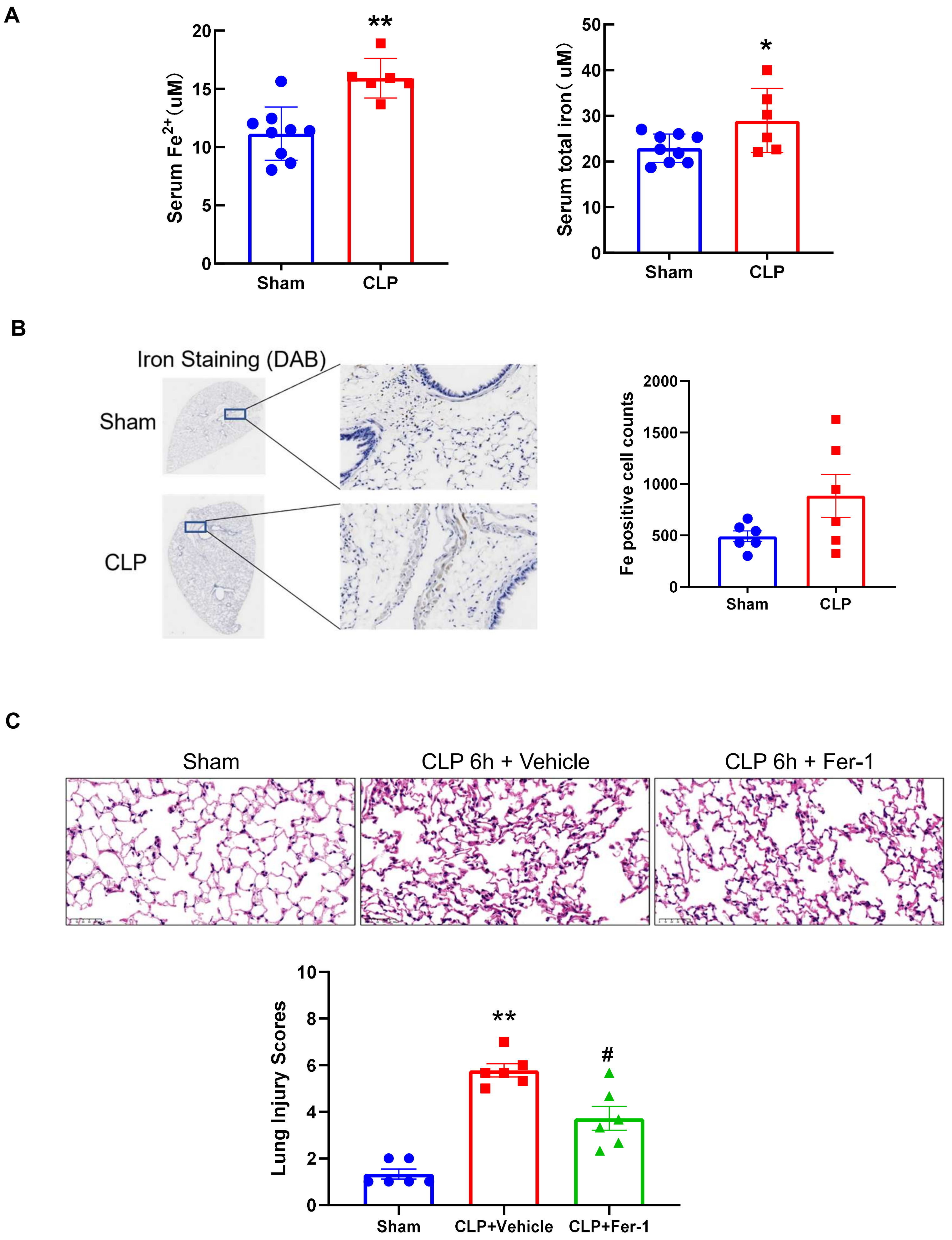

Supplemental Figure 2

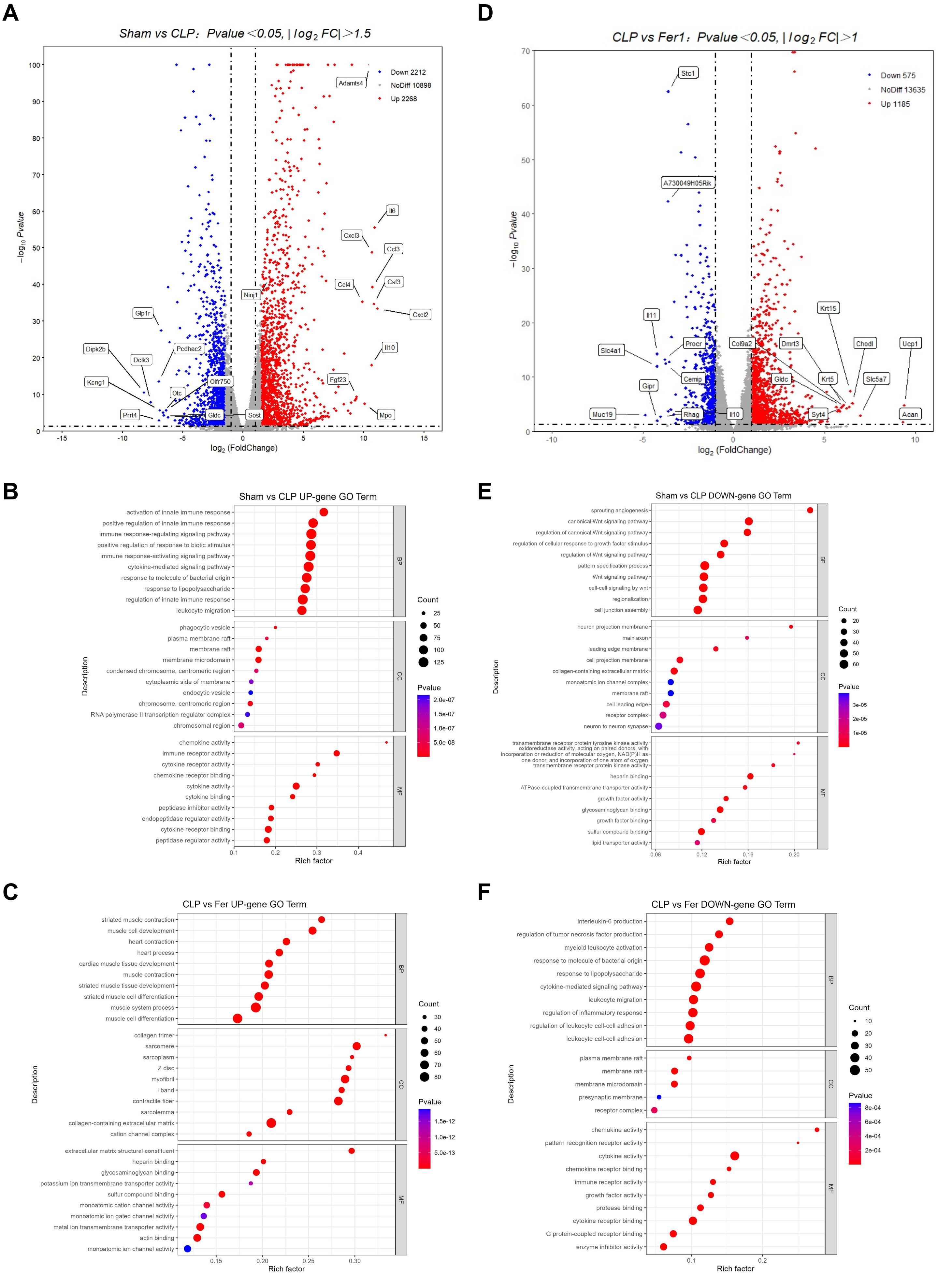

Supplemental Figure 3

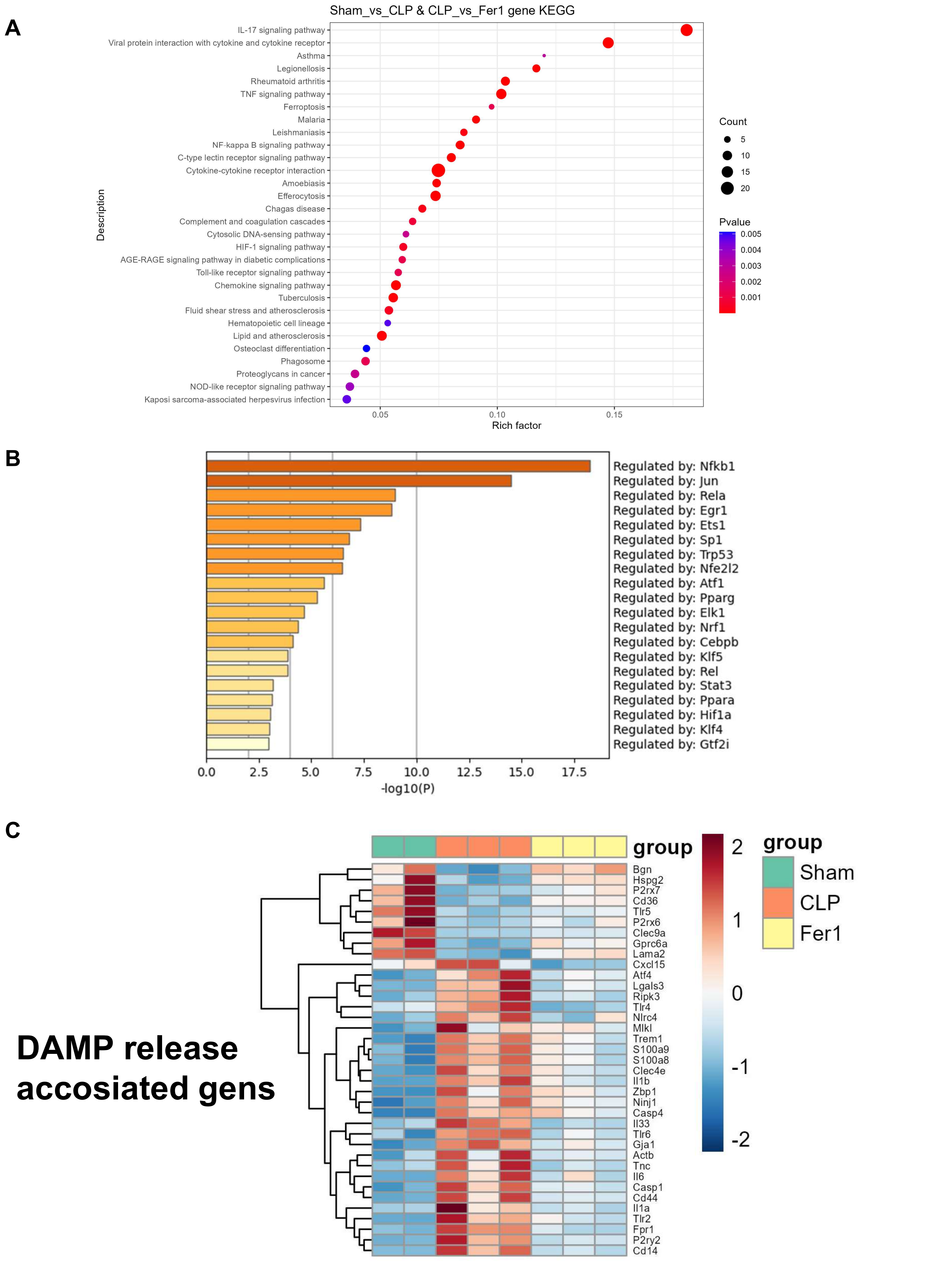

Supplemental Figure 4

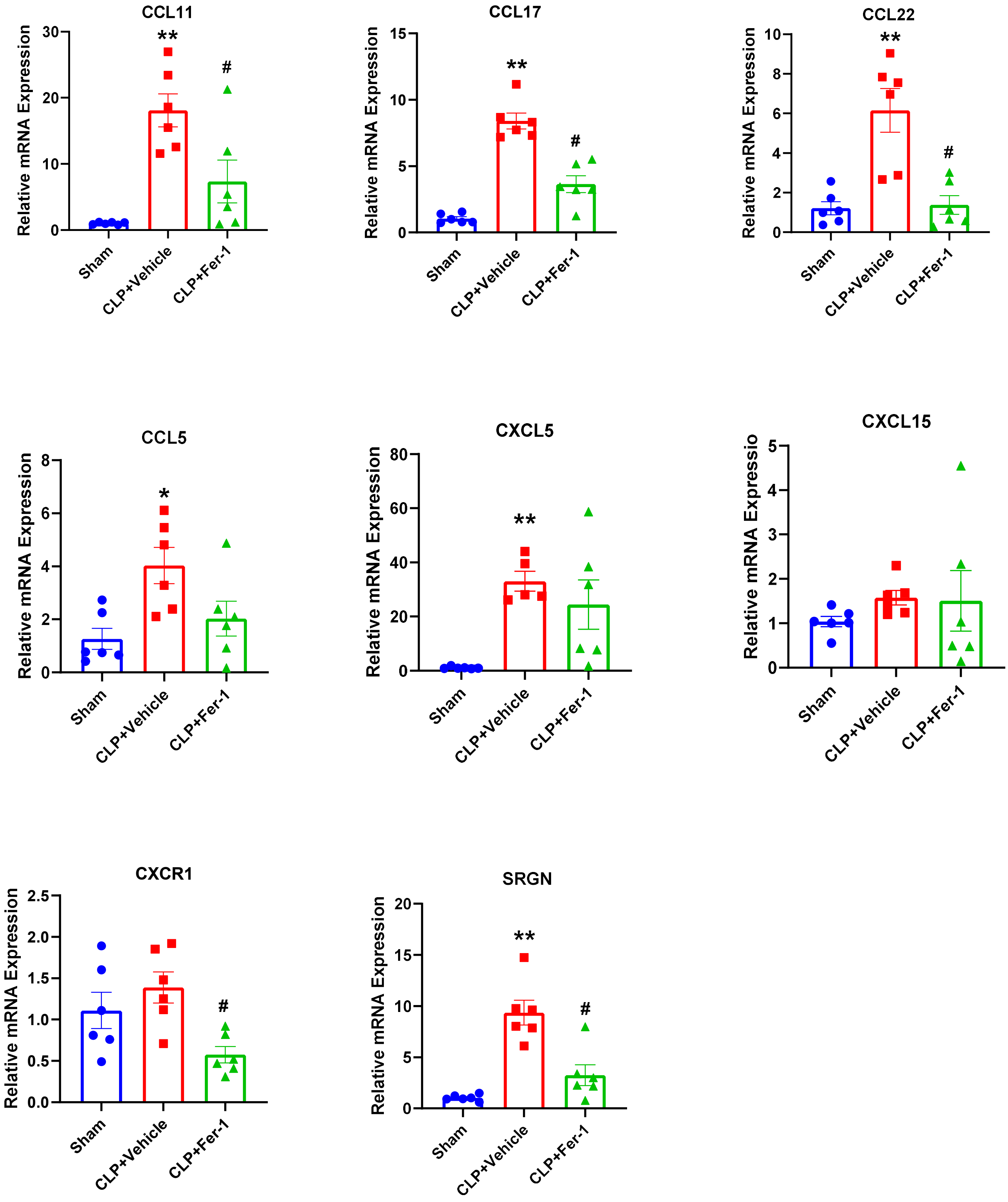

Supplemental Figure 5

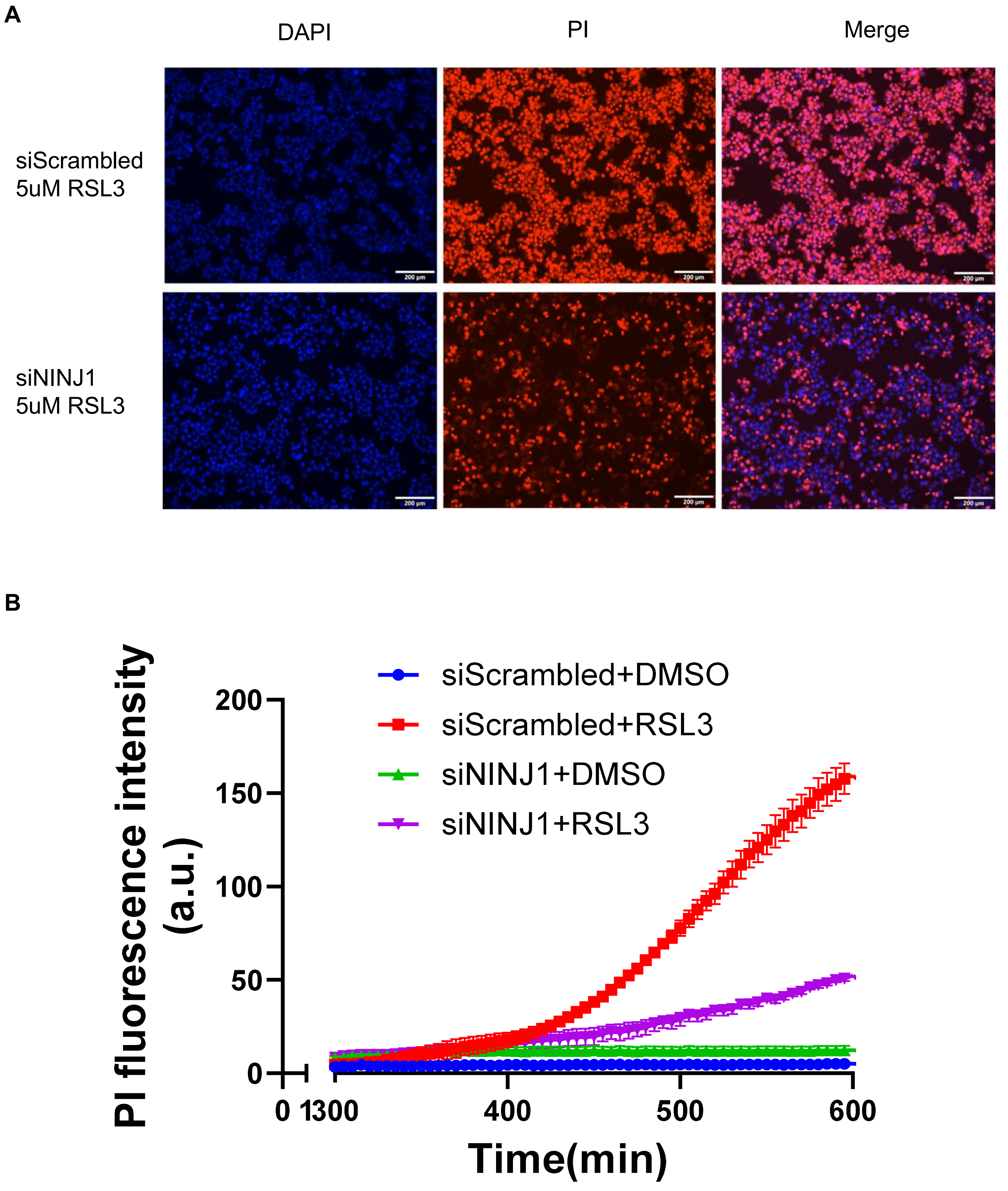

Supplemental Figure 6

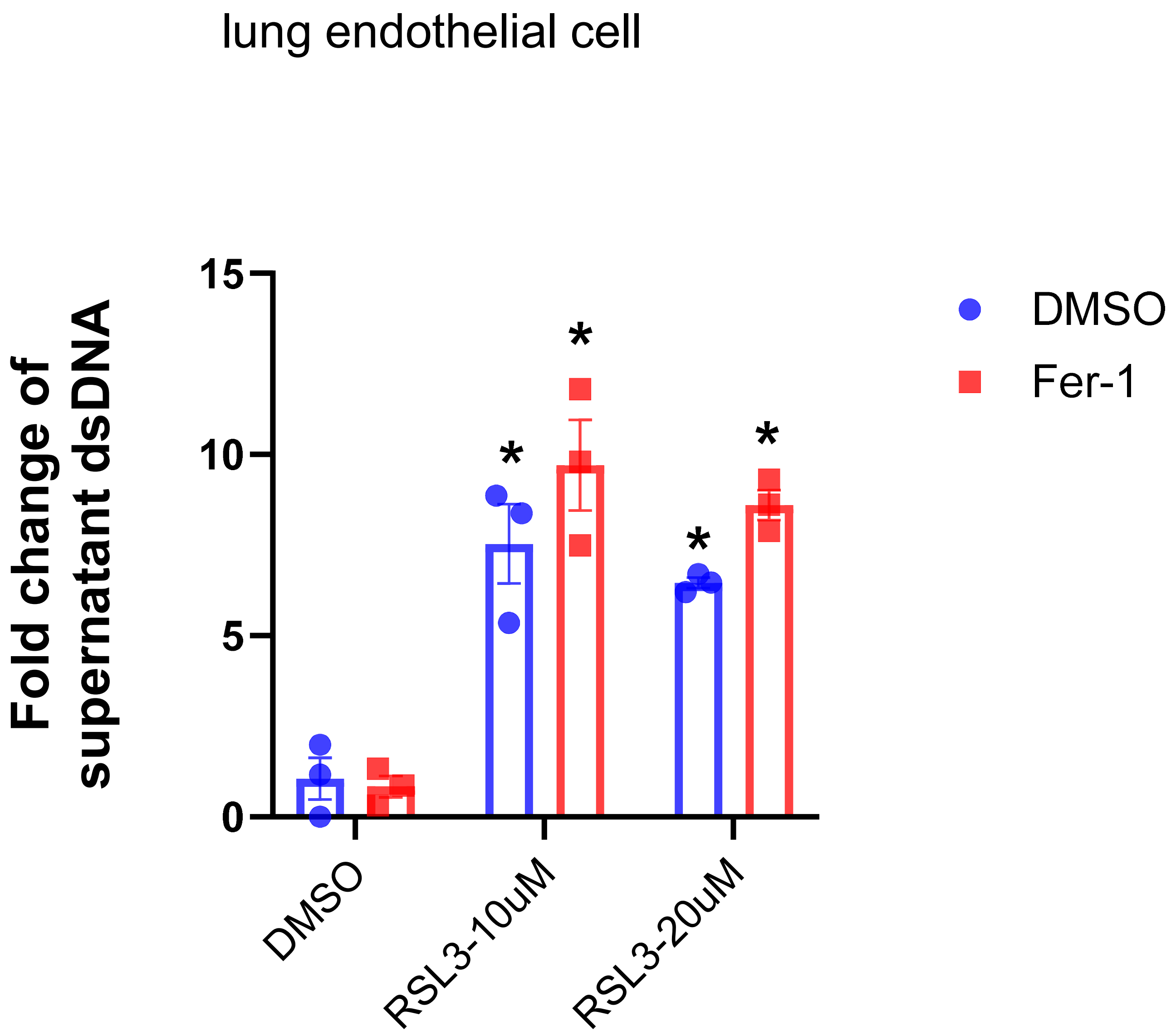

Supplemental Figure 7

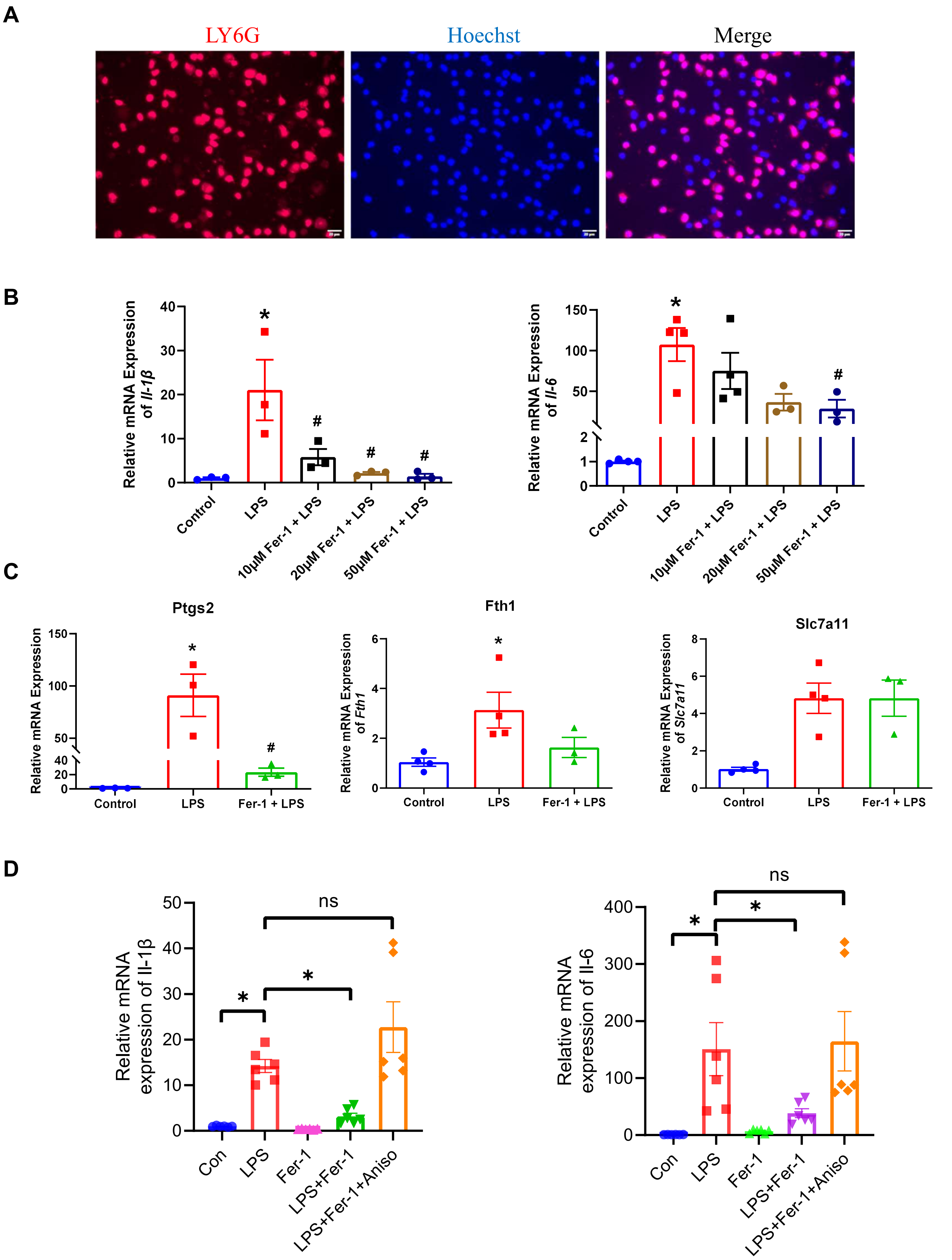

Supplemental Figure 8
